# ACTION-1: study protocol for a randomised controlled trial on ACT-guided heparinization during open abdominal aortic aneurysm repair

**DOI:** 10.1186/s13063-021-05552-7

**Published:** 2021-09-19

**Authors:** Arno M. Wiersema, Liliane C. Roosendaal, Mark J. W. Koelemaij, Jan G. P. Tijssen, Susan van Dieren, Jan D. Blankensteijn, E. Sebastian Debus, Saskia Middeldorp, Jan M. M. Heyligers, Ymke S. Fokma, Michel M. P. J. Reijnen, Vincent Jongkind

**Affiliations:** 1 Department of Vascular Surgery, Dijklander ziekenhuis, Maelsonstraat 3, 1624 NP Hoorn, The Netherlands; 2 Department of Vascular Surgery, Amsterdam UMC, loc. Vrije Universiteit Medical center, De Boelenlaan 1117, 1081 HV Amsterdam, The Netherlands; 3 Department of Vascular Surgery, Amsterdam UMC, loc. AMC, Meibergdreef 9, 1105 AZ Amsterdam, The Netherlands; 4 Emeritus Professor of Clinical Epidemiology & Biostatistics, Department of Cardiology, Amsterdam UMC – University of Amsterdam, 1105 AZ Amsterdam, The Netherlands; 5 Department of Vascular Surgery, University Heart Centre Hamburg-Eppendorf, Martinistrasse 52, 20251 Hamburg, Germany; 6 Division of Internal Medicine, Department of Haematology, Amsterdam UMC, loc. AMC, Meibergdreef 9, 1105 AZ Amsterdam, The Netherlands; 7 Department of Vascular Surgery, Elisabeth-TweeSteden ziekenhuis, Hilvarenbeekseweg 60, 5022 GC Tilburg, The Netherlands; 8 Member of Board of Directors, Dijklander ziekenhuis, Maelsonstraat 3, 1624 NP Hoorn, The Netherlands; 9 Department of Vascular Surgery, Rijnstate ziekenhuis, Wagnerlaan 55, 6815 AD Arnhem, The Netherlands

**Keywords:** Abdominal aortic aneurysm, Open repair, Activated clotting time, Heparin, Vascular surgery, Anticoagulation, RCT

## Abstract

**Background:**

Heparin is used worldwide for 70 years during all non-cardiac arterial procedures (NCAP) to reduce thrombo-embolic complications (TEC). But heparin also increases blood loss causing possible harm for the patient. Heparin has an unpredictable effect in the individual patient. The activated clotting time (ACT) can measure the effect of heparin. Currently, this ACT is not measured during NCAP as the standard of care, contrary to during cardiac interventions, open and endovascular. A RCT will evaluate if ACT-guided heparinization results in less TEC than the current standard: a single bolus of 5000 IU of heparin and no measurements at all. A goal ACT of 200–220 s should be reached during ACT-guided heparinization and this should decrease (mortality caused by) TEC, while not increasing major bleeding complications. This RCT will be executed during open abdominal aortic aneurysm (AAA) surgery, as this is a standardized procedure throughout Europe.

**Methods:**

Seven hundred fifty patients, who will undergo open AAA repair of an aneurysm originating below the superior mesenteric artery, will be randomised in 2 treatment arms: 5000 IU of heparin and no ACT measurements and no additional doses of heparin, or a protocol of 100 IU/kg bolus of heparin and ACT measurements after 5 min, and then every 30 min. The goal ACT is 200–220 s. If the ACT after 5 min is < 180 s, 60 IU/kg will be administered; if the ACT is between 180 and 200 s, 30 IU/kg. If the ACT is > 220 s, no extra heparin is given, and the ACT is measured after 30 min and then the same protocol is applied. The expected incidence for the combined endpoint of TEC and mortality is 19% for the 5000 IU group and 11% for the ACT-guided group.

**Discussion:**

The ACTION-1 trial is an international RCT during open AAA surgery, designed to show superiority of ACT-guided heparinization compared to the current standard of a single bolus of 5000 IU of heparin. A significant reduction in TEC and mortality, without more major bleeding complications, must be proven with a relevant economic benefit.

**Trial registration {2a}:**

NTR NL8421

ClinicalTrials.gov NCT04061798. Registered on 20 August 2019

EudraCT 2018-003393-27

**Trial registration: data set {2b}:**

**Table:** 

Data category	Information
Primary registry and trial identifying number	ClinicalTrials.gov: NCT04061798
Date of registration in primary registry	20-08-2019
Secondary identifying numbers	NTR: NL8421 EudraCT: 2018-003393-27
Source(s) of monetary or material support	ZonMw: The Netherlands Organisation for Health Research and Development Dijklander Ziekenhuis Amsterdam UMC
Primary sponsor	Dijklander Ziekenhuis
Secondary sponsor(s)	N/A
Contact for public queries	A.M. Wiersema, MD, PhD Arno@wiersema.nu 0031-229 208 206
Contact for scientific queries	A.M. Wiersema, MD, PhD Arno@wiersema.nu 0031-229 208 206
Public title	ACT Guided Heparinization During Open Abdominal Aortic Aneurysm Repair (ACTION-1)
Scientific title	ACTION-1: ACT Guided Heparinization During Open Abdominal Aortic Aneurysm Repair, a Randomised Trial
Countries of recruitment	The Netherlands. Soon the recruitment will start in Germany
Health condition(s) or problem(s) studied	Abdominal aortic aneurysm, arterial disease, surgery
Intervention(s)	ACT-guided heparinization 5000 IU of heparin
Key inclusion and exclusion criteria	Ages eligible for the study: ≥18 years Sexes eligible for the study: both Accepts healthy volunteers: no Inclusion criteria:
Study type	Interventional Allocation: randomized Intervention model: parallel assignment Masking: single blind (patient) Primary purpose: treatment Phase IV
Date of first enrolment	March 2020
Target sample size	750
Recruitment status	Recruiting
Primary outcome(s)	The primary efficacy endpoint is 30-day mortality and in-hospital mortality during the same admission. The primary safety endpoint is the incidence of bleeding complications according to E-CABG classification, grade 1 and higher.
Key secondary outcomes	Serious complications as depicted in the Suggested Standards for Reports on Aneurysmal disease: all complications requiring re-operation, longer hospital stay, all complications

**Supplementary Information:**

The online version contains supplementary material available at 10.1186/s13063-021-05552-7.

## Background {6a}

Vascular disease, both occlusive and dilating, is a major contributor to mortality and morbidity. Techniques in both open surgery and endovascular treatments have been refined over the past decades, but at present, they are still associated with mortality and high complication rates [1–8]. Since more than 70 years, unfractionated heparin (further: heparin) is used by all vascular surgeons worldwide during open and endovascular non-cardiac arterial procedures (NCAP), preventing arterial thrombo-embolic complications (TEC) [9–11]. The use of heparin also has a major clinical disadvantage: the prolonged clotting time of blood may increase blood loss, lengthens the time needed for adequate hemostasis, and may cause an increase in bleeding complications. Bleeding complications may require blood transfusions or even surgical (re-)exploration in case of extensive and even life-threatening bleeding. Because of the fine line between thrombosis and bleeding, vascular interventions require precise technique and an accurate, optimal level of coagulation. Another major disadvantage of the use of heparin as a periprocedural prophylactic antithrombotic is the fact that heparin has an unpredictable effect in individual patients [12]. The molecular structure of heparin causes a variety of its effect, creating a difference in efficacy not only between different brands, but even between batches of the same brand [13].

In most countries, heparin is administered as a standardized bolus in every patient undergoing NCAP. The most often used dosage is 5000 IU, irrespective of sex, bodyweight, type of procedure, or duration of procedure. Interventional radiologists often use a dose of less than 5000 IU [9, 10].

In all cardiac interventions worldwide, open or endovascular and using cardio-pulmonary bypass or not, the effect of heparin is measured routinely. Many studies have shown that the activated clotting time (ACT) is the preferred test to measure the effect of heparin and that using this test increases the safety of these cardiac interventions [14, 15]. This results in better patient-related outcomes. Surprisingly, vascular surgeons have not adopted this measurement of the ACT during NCAP. This ACT measurement could ensure the individual patient of safe, tailor-made periprocedural anticoagulation [16–23]. This should lead to better results of procedures, with improved patient-related outcomes and less harm for the patient {6b}.

To evaluate the implementation of routine ACT measurements during NCAP, a prospective registry was instituted in 4 major vascular centers in The Netherlands (MANCO, NTR nr. 6973, ClinicalTrials.gov M016-045). All ACT measurements were performed according to a standardized protocol using the same device: Hemostasis Management System Plus (HMS) by Medtronic®, with high-range ACT cartridges (HR-ACT). The percentage of successful measurements was 99% and results were reproducible and comparable between the different hospitals. The validation and standardization of the HMS for ACT measurements are extensively proven in the literature during cardiac interventions [24, 25]. Similar studies were performed with other cartridges (low-range ACT) for the HMS and other brands of ACT measurement systems. Results (on file, manuscript in preparation) show that the HMS and the HR-ACT guarantee the most stable, reproducible, and comparable results during NCAP. Results of the MANCO study, in more than 700 patients, show that ACT measurements can be introduced safely and adequately in daily routine in the operation room and angio-suite, both during open and endovascular NCAP. Evaluation of these data resulted in a safe and adequate protocol to ensure the patient of optimal, ACT-guided heparinization during NCAP. A goal ACT of 200–220 s is considered to be optimal. A systematic review was conducted by our research group, in which only 4 studies could be found that investigated the relation between ACT values and clinical outcomes [26]. Two studies did not find a relationship between ACT value and bleeding complications [19, 23]. Saw et al. found that an ACT > 300 s was associated with increased combined event rate (death, stroke, or MI) in carotid artery stenting [21]. Kasapis et al. found increased bleeding in peripheral endovascular interventions when the ACT was > 250 s [16].

In the MANCO study, the effect of the standardized bolus of 5000 IU was evaluated by measuring the ACT [27]. Results showed that large individual patient variability in the response to heparin was present. The mean baseline ACT in all patients was 129 ± 18 s and the mean ACT 5 min after the initial bolus of heparin was 191 ± 36 s. After the initial dose of 5000 IU heparin, only 33% and 6% of patients reached an ACT of 200 and 250 s, respectively.

Despite the use of heparin, ATEC occurred in 17 patients (9%). The lowest number of ATEC and hemorrhagic complications occurred in the group of patients with an ACT between 200 and 250 s. Conclusions: A standardized bolus of 5000 IU heparin does not lead to adequate and safe heparinization in non-cardiac arterial procedures. Patient response to heparin shows a large individual variability. Therefore, routine ACT measurements are necessary to ascertain adequate anticoagulation. Further research is needed to investigate if heparin dosing based on the ACT could result in less arterial thrombo-embolic complications, without increasing hemorrhagic complications.

The next step was to design a large international multicenter trial to provide level 1 evidence that ACT-guided heparinization will result in less thrombo-embolic complications, without more bleeding complications than unmonitored heparinization with the use of a standardized bolus. This will be evaluated during open abdominal aortic aneurysm (AAA) surgery DSAA classification C: aneurysm originating below the superior mesenteric artery, DSAA being the Dutch Surgical Aneurysm Audit, a Dutch registration that is mandatory for all Dutch vascular surgeons who treat patients with an AAA [28]. In this registry, details are stored regarding indication, techniques, and periprocedural care. The reason to choose open AAA repair for this RCT is that this procedure is subject to standardized care in all hospitals around Europe, also by following the 2019 European Society of Vascular Surgery Guidelines on Management of Patients with an AAA [29].

During a trajectory of 2 years, funding was applied for at ZorgOnderzoek Nederland Medische Wetenschappen (ZonMw, https://www.zonmw.nl) in close collaboration with the Dutch Surgical Association and Dutch Vascular Surgery board. ZonMw’s principal commissioners are the Dutch Ministry of Public Health, Welfare and Sport (VWS) and the Netherlands Organization for Scientific Research (NWO). ZonMw also increasingly works on behalf of other parties, such as local authorities, health funds, health care insurers, private companies, and professional associations. After an extensive (international) peer-reviewed process, a grant of 1.6 million euros was granted for the ACTION-1 trial: ACT-guided heparinization during open abdominal aortic aneurysm repair.

One of the main demands of ZonMw was to execute a pilot study. Results of this pilot study in 46 patients with open AAA repair resulted in a decrease of TEC from 22% in the 5000 IU group to 7% in the ACT-guided group. No increase in bleeding complications or mortality was detected (no mortality in both groups, E-CABG class 1 bleeding in 39% in the 5000 IU group versus 36% in the ACT-guided group) [30, 31]. In the ACT-guided group, the use of protamine at the end of surgery was also described in a protocol [32, 33]. Because of the limited number of included patients, no statistical significance was reached. This underlines the importance of performing a RCT.

## Method/design

This study protocol has been reported in accordance with the Standard Protocol Items: Recommendations for Clinical Interventional Trials (SPIRIT) guidelines [34].

### Study design {8}

The ACTION-1 trial is a multicenter RCT designed to compare the outcomes of ACT-guided heparinization to a standardized bolus of 5000 IU of heparin, during open AAA repair.

Patients undergoing open AAA repair, meeting eligibility criteria, will be included in the trial after giving written informed consent.

The following Dutch vascular centers (academic and large community training hospitals){9} are currently, or in upcoming months, participating in the ACTION-1 trial: Dijklander Ziekenhuis Hoorn, Amsterdam UMC location VUmc, Amsterdam UMC location AMC, Rijnstate Ziekenhuis Arnhem, Elisabeth-TweeSteden Ziekenhuis Tilburg, Isala Ziekenhuizen location Zwolle, Medisch Spectrum Twente Enschede, Maasstad Ziekenhuis Rotterdam, Groene Hart Ziekenhuis Gouda, St. Antonius Nieuwegein, Alrijne Ziekenhuis Leiderdorp, LUMC Leiden, Amphia Ziekenhuis Breda, Haaglanden MC Den Haag, Gelre Ziekenhuizen Apeldoorn, Slingeland Ziekenhuis Doetinchem, Catharina Ziekenhuis Eindhoven, Zorggroep Twente location Almelo, UMCG Groningen.

Also, the University Heart Center Hamburg is in preparation for intended participation. A website solely for the ACTION-1 study has been developed: ACTION-1.nl. All participating hospitals, inclusion, and information for patients (including lay video) are depicted on this website {9} (Appendix).

The study will be single blinded: only the patient will be fully blinded. Furthermore, the Independent Central Adjudication Committee (ICAC) and the data analysts will be blinded for the intervention {17a}. Blinding will follow all legal demands for unblinding in case of patient safety, as deemed as such by attending medical personnel {17b}. Also, the Data Safety Monitoring Board (DSMB) can decide to unblind.

### Study objectives {7}

To establish that ACT-guided heparinization results in safe and optimal anticoagulation during and thereby less complications after open AAA repair. Domain: complications of treatment. The hypothesis is that ACT-guided heparinization will result in a decrease of TEC and all-cause mortality within 30 days after surgery, without a significant increase in bleeding complications when compared to the use of a non-ACT-guided standardized bolus of 5000 IU. The decrease in TEC will lead to less mortality and morbidity, lower number of re-operations, or better patency, all substantially improving patient’s quality of health, efficiency of medical care, and quality of vascular medical care.

### Sample size calculation {14}

In the DSAA (2014 to 2016), the rate of serious complications was 29% for all patients. According to the Society for Vascular Surgery AAA 2018 guidelines, the incidence of TEC is between 15 and 36%. In our preliminary MANCO trial, the incidence of TEC was 14%. For our power calculation, the incidence of TEC is set at 14%. The vast majority of mortality after open AAA repair stems from TEC. A mortality rate of 5% after open AAA repair is derived from DSAA. The hypothesis is that a decrease of TEC will result in a lower mortality of 3%. Bleeding complications derived from the literature and from our MANCO trial and ACTION pilot study are 18–39% (scored according to E-CABG classification) [30].

Derived from data from our pilot study and from literature, the hypothesis is that ACT-guided heparinization will lower the rate of TEC to 8%. The expected incidence for the combined endpoint of TEC and mortality is therefore set at 19% for the 5000 IU group and 11% for the ACT-guided group. Using a continuity corrected chi-square test with a two-sided alpha of 5%, 337 patients are needed in each group to achieve a power of 80%. Including a dropout of 10%, a total of 750 patients are needed for the combined primary endpoints of TEC and mortality.

In our pilot study, no increase in bleeding complications was found for open AAA repair (E-CABG class 1 bleeding was 39% versus 36%). Nevertheless, it is important that excessive bleeding does not occur in the intervention group. Therefore, a non-inferiority calculation was performed. Bleeding complications and TEC are different and have a different impact on patients. Bleeding complications grade 1 E-CABG have less impact on mortality and quality of life than TEC. The expectation is an improvement in combined TEC and mortality of 8%, and the non-inferiority for bleeding complications is set at 11%.

Expecting 32% bleeding complications in the standard group and 33% in the intervention group and a non-inferiority limit of 43% (11% limit difference) with a power of 80% and a one-sided alpha of 5%, 272 patients are required in each group. Therefore, the 750 patients which are needed for the combined primary endpoints of TEC and mortality are sufficient to also evaluate the non-inferiority for bleeding complications.

### Main study parameter/endpoint efficacy {12}

The combined incidence of all TEC and all-cause mortality within 30 days or during the same admission in hospital, compared between ACT-guided heparinization and a bolus of 5000 IU of heparin. TEC are any complication as caused by thrombus or embolus perioperatively, including but not exclusively myocardial infarction, leg ischemia, deep venous thrombosis, colon ischemia, TIA/stroke, graft thrombosis, peroperative thrombus requiring embolectomy or re-do of an anastomosis, thrombus or embolus in organs or lower limbs, and other peripheral thromboses. The statistical efficacy analysis will be conducted with a chi-square test for proportions. Differences in the incidence of this composite endpoint between the intervention and control groups will be expressed as the absolute risk difference with a 95% confidence interval.

### Main study parameter/endpoint safety {12}

Incidence of bleeding complications according to E-CABG classification, grade 1 and higher: per- or postoperative transfusion of 2 or more units of red blood cells, transfusion of platelets, transfusion of fresh frozen plasma, or re-operation for bleeding during the hospital stay [30, 31]. For the bleeding complications, a non-inferiority test will be used. This will be tested using a one-sided *t* test with an alpha of 0.025.

### Secondary study parameters/endpoints {12}

Secondary endpoints: complications (non-TEC), within 30 days postoperative or in the same admission, as defined by DSAA and suggested standards for reports on aneurysmal disease: all complications requiring re-operation, longer hospital stay, and all other complications; incidence of kidney injury as defined by RIFLE criteria: rise of serum creatinine > 100% or decrease of eGFR with 50% [35]; allergic reactions; ACT values (in the intervention group), total heparin administration, and protamine administration; peroperative blood loss, blood transfusions either autologous or homologous, other blood product administration, total operative time, aortic clamping time, use of adjunctive hemostatic products, and length of hospital (including ICU) stay; health status as measured with the EQ-5D-5L; and economic and health care cost evaluation by IMCQ and IPCQ and addition of out-of-pocket expenses.

### Other study parameters {12}

#### Preoperative parameters

Patient demographics: sex, smoking history, body length and weight and body mass index, medical history (general, cardiac, pulmonary, diabetes, surgical), medication, all previous vascular interventions; blood pressure and pulse at the outpatient visit, ECG reports; diameter and anatomical classification of abdominal or iliac aneurysms; preoperative laboratory results: Hb, leucocytes, sodium, potassium, creatinine, eGFR, platelets; presence of impaired renal function (eGFR < 40 ml/min).

#### Peroperative parameters

Epidural analgesia; surgical approach; clamping sites at arteries.

### Ethical considerations

If patients meet the inclusion criteria, they will be fully informed about the trial and provided with a patient information form and have the opportunity to ask questions. Patients willing to participate will sign the informed consent form. This study will be conducted in accordance with the Good Clinical Practice (GCP) guidelines, with the principles of the Declaration of Helsinki, and with the Medical Research Involving Human Subjects Act (WMO). The medical ethical committee in Amsterdam (2019.732 – NL6675902919) has approved the study protocol, as well as local institutional boards of each participating center. All legal European demands concerning insurances for possible harm from trial participation are met and all separate trial study sites have insurance as legally demanded by the Dutch Government for non-trial harm for participating patients {30}.

### Safety and quality control

#### Independent Central Adjudication Committee

The ICAC is instituted to decide whether complications are rightfully labeled as TEC in the CRF. Two vascular surgeons and 1 registered intensive care specialist will form this committee, none of them being a member of the ACTION-1 project group.

This committee will gather 30 days after 100, 200, and 500 inclusions and 6 weeks after the last inclusion. They are blinded for the intervention and will judge the complication parts of the CRFs of all included patients.

In case of disagreement within this committee, the majority will be decisive. In case this committee decides that they need further clarification on a specific complication, this will be provided by the project group with data from the original electronic patient file of the patient.

### Data Safety Monitoring Board {21 a,b}

Despite the fact that this study is labeled as moderate risk, a full DSMB is installed. The DSMB is composed of three independent experts: a vascular surgeon, a cardio-thoracic surgeon, and a clinical epidemiologist and biostatistician.

A safety review will be performed by an independent statistician (T. van der Ploeg, PhD) and reviewed by the data safety monitoring committee after the results are available for 100, 200, and 500 patients. This is a safety review, which looks at the combination of several outcomes as opposed to a traditional interim analysis with specified stopping rules.

In case of strong concerns about safety, the safety monitoring committee can advise to stop the study. Furthermore, serious adverse events (SAEs) will be reported to the data and safety monitoring committee.

A total of three safety reviews are planned:

- A first interim analysis is planned when approximately 100 subjects have been enrolled. This will provide data sample size calculations and safety assessments.
- A second interim analysis is planned when approximately 200 subjects have been enrolled. This will provide data sample size calculations and safety assessments.
- A third interim analysis is planned when approximately 500 subjects have been enrolled. This will provide data sample size calculations and safety assessments.

Additional ad hoc interim analyses may be conducted to support decision-making concerning the current clinical study, the sponsor’s clinical development projects in general or in case of any safety concerns.

Independent personnel who are not directly involved in conducting the study will perform the interim analyses and review of the unblinded outputs.

The DSMB should consider stopping the study if the following conditions are met:

The stopping rule for safety is:

- A difference in all-cause mortality within 30 days after surgery or during the same admission between intervention and control groups with *P* value smaller than 0.05 in disadvantage of the intervention group
- A difference in life-threatening bleeding (E-CABG classification grade 2 or higher: transfusion of 5 or more units of red blood cells or re-operation for bleeding) between the intervention and control groups with a *P* value smaller than 0.05 in disadvantage of the intervention group
- A difference in the composite of all-cause mortality or life-threatening bleeding (E-CABG classification grade 2 or higher: transfusion of 5 or more units of red blood cells or re-operation for bleeding) between the intervention and control groups with a *P* value smaller than 0.05 in disadvantage of the intervention group

Stopping rules for efficacy:

The DSMB should only under exceptional circumstances advise to terminate the trial under overwhelming efficacy of the ACT-guided heparin group over the control group: the DSMB could consider stopping when a difference in the incidence of TEC and mortality within 30 days after surgery or during the same admission between the intervention and control groups with a *P* value smaller than 0.001 occurs, according to Haybittle–Peto boundary.

No further dissemination of interim results should occur, in particular not with individuals involved in treating the study’s subjects or assessing clinical data.

While monitoring guidelines have been provided, the DSMB uses all available evidence and its collective judgment to base its recommendation to stop or modify the study.

### Adverse, severe adverse events, and suspected unexpected serious adverse reactions {22}

Adverse events are defined as any undesirable experience occurring to a subject during the study, whether or not considered related to the intervention. All adverse events, within 30 days postoperative or in the same admission, reported spontaneously by the subject or observed by the investigator will be recorded. During all procedures in ACTION-1, one of the trained team members will be present in the operating room during the duration of the entire procedure. This team member will complete the peroperative variables in the electronic CRF. After 30 days, the electronic patient file will be checked for complications, and the eCRF will be completed. All AEs and SAEs will be systematically recorded.

A SAE is defined as any untoward medical occurrence or effect that results in death, is life threatening (at the time of the event), requires hospitalization or prolongation of existing inpatients’ hospitalization, and results in persistent or significant disability or incapacity, or any other important medical event that did not result in any of the outcomes listed above due to medical or surgical intervention but could have been based upon appropriate judgment by the investigator. An elective hospital admission will not be considered a serious adverse event.

All SAE will be reported by the local principal investigator to the sponsor within 24 h of the study site staff becoming aware of the event. The sponsor will report all the SAE in a line listing, which will be reported once every 6 months to the medical ethical committee.

Adverse reactions are all untoward and unintended responses to an investigational product related to any dose administered. Unexpected adverse reactions are suspected unexpected serious adverse reactions (SUSARs) if the following three conditions are met: the event must be serious; there must be a certain degree of probability that the event is a harmful and an undesirable reaction to the medicinal product under investigation, regardless of the administered dose; and the adverse reaction must be unexpected, that is to say, the nature and severity of the adverse reaction are not in agreement with the product information as recorded in the summary of product characteristics.

All SUSARs will be reported by the sponsor to the DSMB and to the accredited medical ethical committee via “Toetsingonline” on the website of the Central Committee on Research involving Human Subjects (CCMO, www.ccmo.nl).

Independent monitoring and extensive quality control including all extensive legal demands for major trials with pharmaceuticals are met by an international acclaimed bureau: Julius Clinical (https://www.juliusclinical.com).

All pre-specified AEs and SAEs will be collected and reported in trial publications.

### Inclusion criteria {10}

Inclusion criteria are able to speak and read in the local language of the trial hospital; patients older than 18 years scheduled for elective, open repair of an iliac or abdominal aortic aneurysm distal of the SMA (DSAA segment C); implantation of a tube or bifurcation prosthesis; trans-abdominal or retroperitoneal surgical approach of aneurysm; and able and willing to provide written informed consent.

### Exclusion criteria {10}

Exclusion criteria are not able to provide written informed consent; previous open or endovascular intervention on the abdominal aorta (previous surgery on other parts of the aorta or iliac arteries is not an exclusion criterion); history of coagulation disorders, heparin-induced thrombocytopenia (HIT), allergy for heparin or thrombocyte pathology; impaired renal function with EGFR below 30 ml/min; acute open AAA surgery; hybrid interventions; connective tissue disorders; dual anti-platelet therapy, which cannot be discontinued; life expectancy less than 2 years; inflammatory, mycotic, or infected aneurysms; and allergy for protamine or fish protein.

### Recruitment {15}

**Fig. 1:**
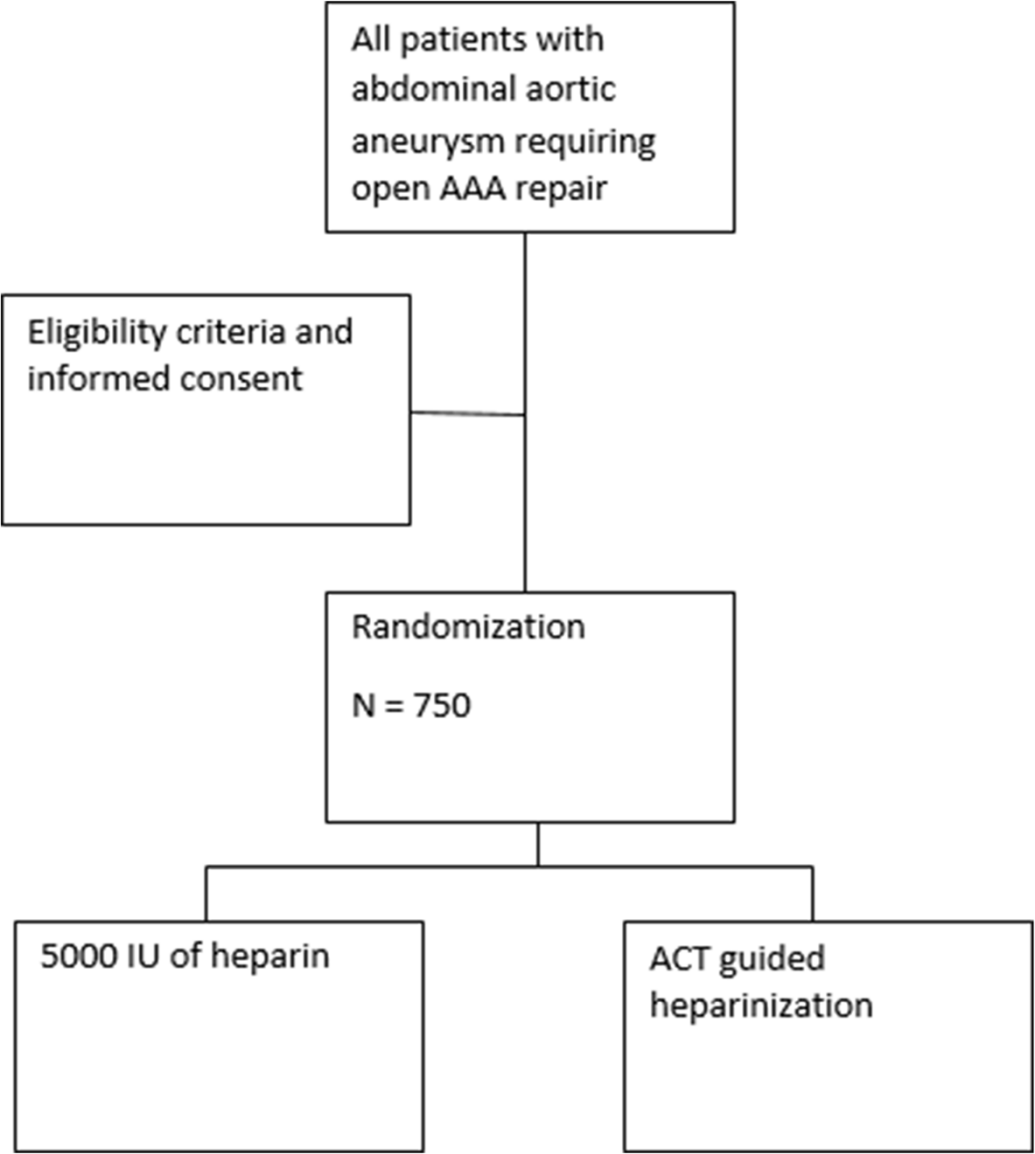
Recruitment

**Fig. 2:**
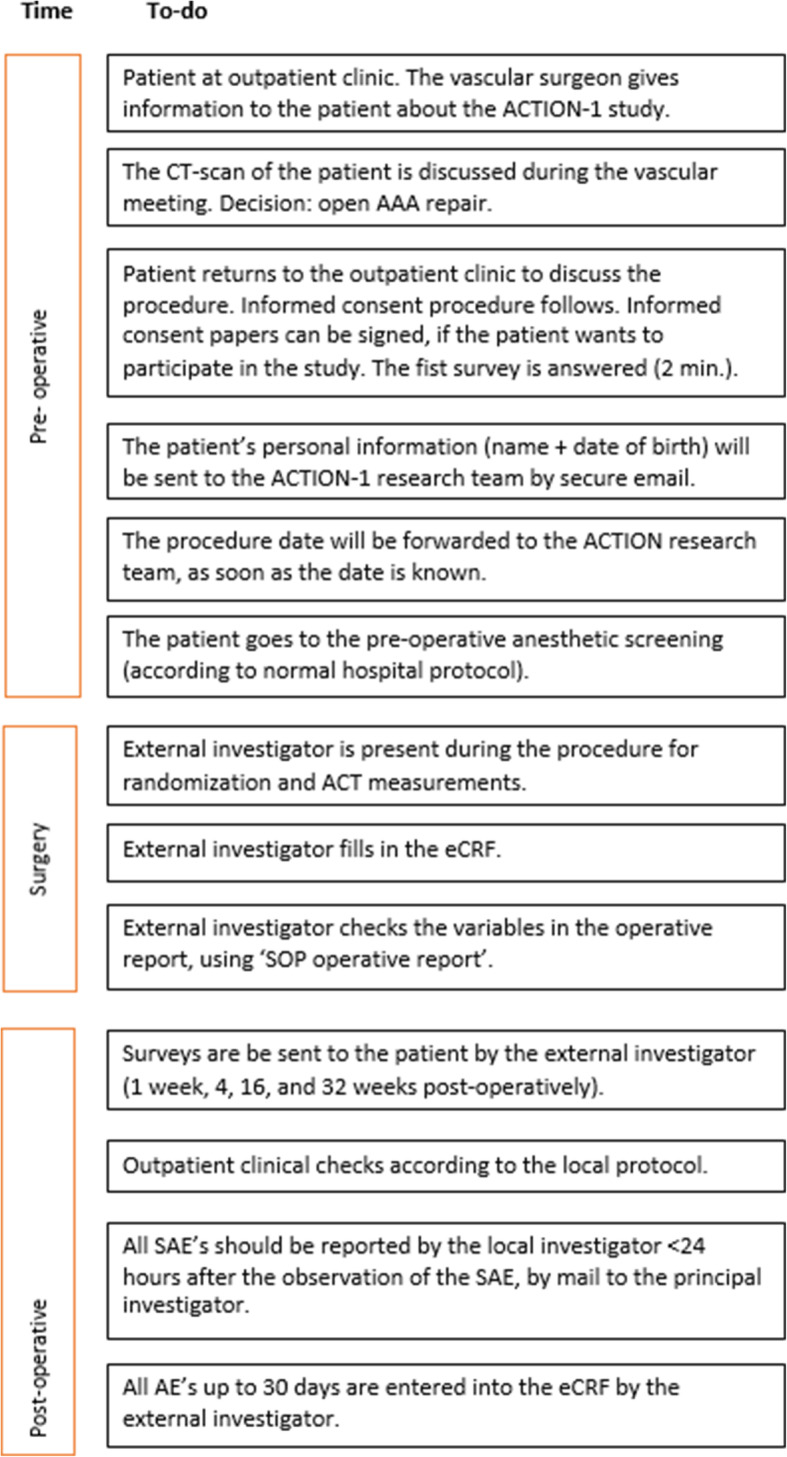
Participant timeline

**Fig. 3:**
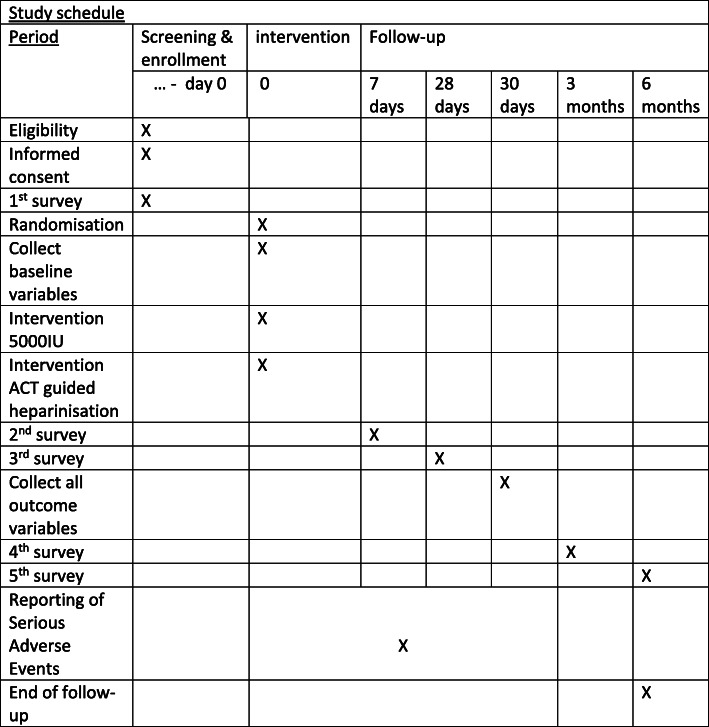
Schedule of enrollment, interventions, and assessment

Patients scheduled to undergo open AAA repair will be informed about the study by their attending vascular surgeon in the outpatient clinic of participating hospitals about the study and the informed consent procedure will be explained. Informed consent will only be obtained by medical personnel who are GCP licensed. Also, a mandatory training by research staff has to be completed and the site initiation visit (SIV) completed {26a}. This SIV is performed by an external, independent trial research organization: Julius Clinical. A total of 750 patients with an abdominal aortic aneurysm requiring open aneurysm repair will be included in the ACTION-1 study, after signing informed consent (Fig. 1). An EQ-5D-5L questionnaire is handed out to the patient after receiving informed consent. The patient returns the form by post to the investigators, or bring the form when admitted for surgery, for baseline values. Figure 2 shows the participant timeline {13} and Fig. 3 shows the study schedule.

### Randomization {16 a,b,c}

Randomization will take place just before the start of surgery by one of the researchers of the sponsor, who is present in the operating room during the entire procedure. A computerized program (CASTOR EDC) is used with a random block size of 2, 4, and 6. The randomization will be stratified by participating center. The assigned group (ACT or 5000 IU) is displayed on the computer, immediately after randomization. The researcher will inform the anesthesiologist which dose of heparin should be administered.

### Treatment details {11a}

#### ACT-guided heparinization

Heparin is given to reach an ACT of 200–220 s. At the start of the procedure, before any heparin is given, a baseline ACT measurement is performed. Three to 5 min before clamping of the aorta, 100 IU/kg bodyweight of heparin is administrated intravenously. If patients weigh more than 150 kg, a maximum heparin dose of 15,000 IU heparin is administered to prevent overdose.

Five minutes after administration of heparin, ACT measurement is performed. If the ACT is below 180 s, an additional dose of heparin of 60 IU/kg is administered. If the ACT is between 180 and 200 s, an additional dose of heparin of 30 IU/kg is administered, and if the ACT is 200 s or longer, no extra heparin is given.

Five minutes after every administration of heparin, the ACT is measured. If the ACT is 200 s or longer, the next ACT measurement is performed every 30 min, until the end of the procedure or until new heparin administration is required (because of ACT < 200 s). After each new dose of heparin, an ACT measurement is performed after 5 min and the above-described protocol of ACT measurements will be repeated. After re-establishing blood flow and removing all clamps, the ACT is measured. Depending on that ACT value near the end of the surgery, protamine is given to neutralize the effect of heparin.

If the ACT at closure is between 200 and 250 s, 2500 IU protamine should be administered. If the ACT is higher than 250 s, 5000 IU protamine should be administered, and if between 180 and 200 s, 1000 IU protamine. Five minutes after the administration of protamine, the ACT is measured. The ACT should preferably be below 180 s. If the ACT is still more than 200 s, protamine should be administered again using the above-mentioned protocol. When an additional dose of protamine is required, ACT measurement is performed 5 min after that administration.

#### 5000 IU of heparin

A single dose of 5000 IU of heparin is given 3–5 min before clamping of the aorta. No ACT measurements are performed. Only on clarified indications, extra doses of heparin or protamine are permitted, at the discretion of the attending vascular surgeon. Deviations from the protocol will be clearly stated with reasoning in the operative report.

Patients with additional doses of heparin or protamine outside the protocol will not be excluded from the trial. Evaluation will be performed according to intention-to-treat analysis but also a per-protocol analysis will be performed and, if indicated, a sensitivity analysis.

In the intention-to-treat analysis, all patients who were randomised and meet the inclusion and exclusion criteria will be included.

A per-protocol analysis will be performed on the patients who fulfill the following:

ACT group:

1. Patients who received a starting dose of 100 IU/kg and reached an ACT ≥200 s within 30 min after cross clamping of the aorta with or without additional heparin dosages

and

2. Received extra dosage(s) of heparin in case ACT dropped below 200 s during cross clamping (noncompliance in case of restoration of arterial blood flow within 10 min is allowed)
3. ACT at the end of surgery was <180 s with or without administration of protamine

5000 IU group:

The patient received a start dose of 5000 IU of heparin.

Patients will be excluded from the per-protocol analysis if:

ACT group:

- Patients who did not receive a starting dose of heparin of 100 IU/kg
- Patients who did not reach an ACT > 200 s within 30 min after cross clamping of the aorta
- Patients who received extra heparin despite ACT > 200 s during cross clamping
- Patients who received protamine despite ACT < 180 after restoration of arterial blood flow
- Patients where the administered dose of heparin or protamine deviates from the directions in the protocol with more than 50% (upper or lower)

5000 IU group:

- Patients who did not receive a starting dose of heparin 5000 IU

Patients will not be transferred to the other group.

### Follow-up and quality of life measurements

Postoperative treatment, blood tests, and outpatient clinic visits will be according to local protocols. The patients will be sent 3 kinds of surveys: the EQ-5D-5L for quality of life evaluation, after 1 week, 4 weeks, 16 weeks, and 23 weeks postoperatively; the iMCQ, for the evaluation of medical consumption, after 23 and 26 weeks postoperatively; and the iPCQ, for the economic evaluation, after 26 weeks postoperatively. These forms can be completed online or at home by the patients and send to the investigators by post. These questionnaires will be included in the CRFs

### Data collection and management {18 a,b, 19}

After 30 days, all postoperative variables will be collected into the electronic database. All study parameters are standard care and can be reproduced from electronic patient files. Extensive standard operating procedures (SOP) are present to secure that data is properly scored.

All data will be collected at each participating center using the eCRF in the electronic database Castor EDC. Castor complies with all applicable laws and regulations with regard to ICHG GCP and the General Data Protection Regulation (GDPR). Each participating center will maintain a key list. This key list stays in the local hospital and will not be shared. After completion of the study, all study documents will be stored on site for 25 years.

All participating sites will be monitored by a Clinical Research Associate (CRA) of the sponsor Dijklander ziekenhuis and a selection site by a monitor of Julius Clinical, a Clinical Research Organization (CRO) {27}.

After completion of the trial, all raw data will be made available for others, following the mandatory policy of ZonMw. No contractual agreements are made that limit any access for other investigators {29}.

### Statistical analysis {20 a,b,c}

Descriptive statistics of continuous variables will be presented as means with standard deviations (SD) or medians with inter-quartile ranges (IQR) depending on the distribution of the data.

Categorical data will be presented as proportions and numbers. The statistical efficacy analysis will be conducted according to the intention-to-treat principle. A separate per-protocol analysis will be performed additionally as a sensitivity analysis. All analyses will be performed with the latest version of the Statistical Package for Social Sciences (SPSS, SPSS Inc., Armonk, NY, USA).

The analysis of primary efficacy and safety outcomes will be performed on an intention-to-treat basis and in a hierarchical fashion. If the statistical analysis shows that there is a statistically significant difference in the primary efficacy endpoint, a statistical analysis of the primary safety study parameter will be performed. If there is no significant difference between study groups in primary efficacy endpoint, assessment of primary safety endpoint will be considered exploratory. Subjects with missing data will be excluded per analysis.

### Primary efficacy study parameter

The primary endpoint is the composite of the incidence of all TEC, including myocardial infarction, leg ischemia, deep venous thrombosis, colon ischemia, stroke, graft thrombosis, thrombo-embolic complications in the kidney or spleen and other peripheral thromboses and all-cause mortality within 30 days after surgery or during the same admission, and peroperative thrombosis requiring additional actions peroperatively (i.e., embolectomy, atherectomy, or re-do of an anastomosis because of thrombus). The statistical efficacy analysis will be conducted with a chi-square test for proportions. Differences in the incidence of this composite endpoint between the intervention and control groups will be expressed as the absolute risk difference with a 95% confidence interval.

### Primary safety study parameter

Incidence of bleeding complications according to E-CABG classification, grade 1 and higher [31]. For the bleeding complications, a non-inferiority test will be used. We test the hypothesis that the difference in bleeding between the intervention group and the control group is below the a priori specified boundary of 11%. This will be tested using a one-sided *t* test with an alpha of 0.025, with the null hypothesis that the number of bleedings is above the threshold margin and the alternative hypothesis that is below the threshold margin. If the confidence interval for the bleeding complications does not include the non-inferiority limit in the per-protocol analysis and in the intention-to-treat analysis, non-inferiority for bleeding complications is established.

### Secondary study parameter(s)

Secondary endpoints include all complications as defined by DSAA and suggested standards for reports on aneurysmal disease. Health status was measured with the EQ-5D-5L questionnaire. Differences in categorical outcomes between the intervention and control groups will be expressed as the absolute risk difference with a 95% confidence interval. Differences in continuous outcomes will be tested with Student’s *t*-test in case of a normal distribution or the Mann-Whitney *U*-test in case the data do not follow the normal distribution. The level of significance is set at a two-sided *P*-value < 0.05.

### Other study parameters

Peroperative blood loss (continue), blood transfusions either autologous or homologous (categorical), other blood product administration (categorical), total operative time (continue), clamping time (continue), use of adjunctive hemostatic products (categorical), length of hospital (including ICU) stay (continue), and health status (categorical and continue); ACT values measured (continue); the amount of heparin and protamine used (categorical). The outcomes of the first 5 patients from all participating hospitals will be analyzed and compared with the outcomes of patients included later. Data on the previous heparin protocol will be collected per hospital. Analyses will be conducted to determine whether the previously used heparin protocol affects the outcomes.

## Economic evaluation

### Cost-effectiveness analysis (CEA)

General considerations: We hypothesize that ACT-guided heparinization could lower the rate of TEC and TEC-related mortality to in total 11% and that the quality of life can be increased from 73 to 76%. The economic evaluation of ACT-guided heparinization against standard care with a standardized bolus of heparin will be performed as cost-utility analyses and a cost-effectiveness analysis from a societal perspective with the costs per quality-adjusted life year (QALY) and the costs per prevented complication as the primary economic outcomes. The cost-utility analysis can be used for policy making and composition of a guideline. The cost-effectiveness analysis (CEA) relates to the clinical outcome parameter and may be used for prioritization or benchmarking of strategies that enhance surgical patient safety. The CEA and CUA will be based on a time horizon of 6 months. All related complications are within the time horizon of 6 months and patients will be recovered from the surgery. For on-going complications such as leg amputations, colostomy, permanent neurological deficits, and dialysis, a CEA and CUA with a lifelong time horizon will be made using extrapolation and model-based techniques. For this time horizon, discounting of effects and costs will be performed as stated in the most recent guidelines for cost analysis [36]. To account for uncertainties in the lifelong time horizon, a probabilistic sensitivity analysis will be performed.

Incremental cost-effectiveness ratios will be calculated as the difference in costs per QALY gained and as the difference in costs prevented complications. Sampling variability will be accounted for by bias-corrected and accelerated non-parametric bootstrapping. Results will be reported along with their 95% confidence intervals and displayed graphically with cost-effectiveness planes and with cost-effectiveness acceptability curves. One-way and multi-way sensitivity analyses will be done for the unit costs of the most common complications. Some missing data can be expected; if missing data is at random, this will be handled through multiple imputations with predictive mean matching.

### Cost analysis

Medical costs, patient costs, and productivity losses will be included in the evaluation. The medical costs cover the costs of surgery and related complications, anesthesia, theater, perioperative materials, inpatient stay at the ICU, and the wards and medications. The patient costs include out-of-the-pocket expenses like over-the-counter medication and health care-related travel costs. Productivity losses are costs resulting from being absent and decreased productivity during work. Hospital health care utilization will be retrieved from CRFs and hospital information systems. Data on out-of-hospital health care will be gathered with the iMTA Medical Consumption Questionnaire (iMCQ) adjusted to the study setting. The productivity losses will be documented with the iMTA Productivity Cost Questionnaire (iPCQ). Questions on out-of-pocket expenses will be added to these patient questionnaires. Costs will be price indexed based on consumer price indices (CPI).

Costs will be calculated for individual patients as the product sum of the resource use and the respective unit costs. The iMCQ questionnaire will be sent 13 and 26 weeks after surgery, and the iPCQ only 26 weeks after surgery.

### Patient outcome analysis

Patients will be asked to complete the EQ-5D-5L health status questionnaire at baseline, 1 week, 4 weeks, 13weeks, and 26 weeks after surgery. These forms can be completed online or at home by the patients and send to the investigators by post. These questionnaires will be included in the CRFs. The EQ-5D-5L scoring profiles can be converted into a health utility score based on general population-based Dutch tariffs [37]. QALYs will be calculated for each patient using linear interpolation between the successive health utility assessments over time.

### Publication of data {31a,b,c}

During the informed consent procedure, participants can indicate whether they want to be informed about the results of the study. The results will be shared after the last patient completed the 6-month surveys. Results will also be published in a peer-reviewed journal and will be described on clinicaltrials.gov.

Persons with substantive contributions to the design, conduct, interpretation, and reporting of this trial will be recognized through the granting of authorship on the final trial report.

The participant-level dataset will be shared under pre-defined conditions and contract.

## Discussion

The ACTION-1 trial is conducted to investigate if ACT-guided heparinization might lead to better (patient related) outcomes than a standardized bolus of 5000 IU of heparin without measuring its effect. The trial will be executed during open AAA repair in 18 large Dutch Vascular Centers (University and non-University) and 2 major centers in Germany and Denmark.

One of the possible concerns on operational issues might be the inclusion rate. Although the incidence of open AAA repair has declined considerably during the past decades due to the “EVAR first” policy, a stabilization or even small increase in open AAA repair is present. The much-discussed recent NICE Guidelines on AAA treatment and the strong recommendation issued by the Dutch Board of Vascular Surgery to perform EVAR within the applicable IFU could be contributing factors to the renewed focus on open repair [38]. Apart from the inclusion issue, some vascular surgeons may experience “cold feet” when their patient is randomized to an arm of the study that is not their preferred heparin regimen. Although our study group has proven convincingly that no evidence is present on either 5000 IU or ACT-guided heparinization, the strong belief and year-long routine of the individual surgeon can be hard to put aside [26, 27]. Therefore, it might be anticipated that protocol deviations could occur on this aspect. For example, the surgeon not administering a second dose of heparin if ACT is below 200 s in the ACT group, or an extra gift of heparin outside the protocol if the patient is randomized in the 5000 IU group. The frequency of this reluctance to adhere to the protocol is deemed to be low and equal in both groups. Before the definite participation of each vascular center, a 30-min presentation and discussion was held in which it was underlined that no evidence is present on either heparin regimen. Also, the strong support of the Board of Dutch Vascular Surgeons and the Board of Dutch Medical Specialists contributes to creating equipoise among participating surgeons. To further enhance this feeling, it is emphasized in the protocol that individual surgeons are allowed to deviate from the protocol if this is deemed necessary for patient safety. Furthermore, during all procedures in ACTION-1, one of the trained team members will be present in the operating room during the duration of the entire procedure. The team member will randomize the patient when anesthesia is completed and the team member will perform all ACT measurements, if applicable, to exclude as much as possible any incorrect measurements or inconsistencies regarding ACT measurements. Also, the attending team member will record all variables present in the eCRF. In this manner, maximal exclusion of bias can be achieved. During the 2 years of finetuning the protocol for ACTION-1 and in the process of extensive, repeated international peer-reviewing by the funding agency ZonMw, all possible protocol and operational issues were discussed and, hopefully, anticipated.

Due to the COVID-19 pandemic, access to the participating hospitals was limited for visitors. Therefore, the presentations about the ACTION-1 trial and the site initiation visits were postponed. In addition, many COVID-19 patients were admitted to the intensive care unit, limiting the capacity for patients after open AAA surgery. As a result, less patients were operated for an AAA and thus not included in the ACTION-1 trial. No changes were needed in the protocol because of COVID-19.

## Trial status

Medical Ethics Committee and CCMO approval was obtained on the 21st of February 2020 {24}. The current protocol of the ACTION-1 study is version 12.2, 21-07-2021 {3}. All major protocol modifications and amendments will be submitted to the Medical Ethics Committee, shared with the participating hospitals, and published on clinicaltrials.gov. The recruitment of the study began in March 2020. At the current date, 90 patients have been included already despite the delay in the preparation of participating hospitals due to the COVID-19 pandemic. The completion of the study is expected in December 2024, with a 6-month extension period granted by ZonMw due to the COVID-19 pandemic.

### Protocol version {3}

Protocol ID: NL-6675902919 Version 12.2 dd. 21-07-2021

### Funding {4}

Financial: ZonMw grant: 848043004

In-kind: Medtronic® for contribution for Hemostasis Management System Plus devices

In-kind: Dijklander ZH, Amsterdam UMCs: personnel

### Roles and responsibilities {5a}

Design, protocol development, application grant ZonMw: AW, MR, JB, MK, SM, SvD, JT, and VJ

Finetuning protocol, different aspects: LR, YF, ED, and JH

### Trial sponsor {5b}

Dijklander ziekenhuis, Raad van Bestuur (Board of Directors)

Y.S. Fokma, MSc

Mealsonstraat 3, 1624 NP Hoorn, The Netherlands

0031-229257257

### Role of the study sponsor and funders {5c}

No influence on data collection management, analysis and interpretation of data, writing the report, and the decision to submit the report for publication, including no ultimate authority over any of these activities.

### Role and composition and responsibilities of the coordinating center and other committees {5d}

PI and Dijklander ziekenhuis will have end responsibility according to all legal demands. Furthermore, all legal demands are met with independent trail supervision and extensive control by Julius Clinical. Independent Central Adjudication Committee (ICAC): this ICAC is instituted to decide whether complications are rightfully labeled as thrombo-embolic complication (TEC) in the case report form (CRF). Data Safety Monitoring Board (DSMB) is fully instituted according to the highest level of legal safety demands. See protocol for detailed responsibilities.

### Supplementary Information

**Additional file 1:.** Ethical approval document, official English summary included.

**Additional file 2:.** Original funding documentation.

**Additional file 3:.** English translation of original funding documentation.

**Additional file 4:.** SPIRIT 2013 Checklist: Recommended items to address in a clinical trial protocol and related documents.

## Data Availability

The participant-level dataset will be shared under pre-defined conditions and contract.

## Appendix

{32}: Model consent form and other related documentation given to participants and authorized surrogates. Patients and physicians can find information about the ACTION trial also on ACTION-1.nl. All participating hospitals, inclusion, information for patients (including lay video) are depicted on this website.

{33}: No biological specimens are stored.

## Abbreviations

AAA: Abdominal aortic aneurysm
ACT: Activated clotting time
CCMO: Central committee on research involving human subjects
CRA: Clinical research associate
CRO: Clinical research organization
CRF: Case report form
DSAA classification C: Aneurysm originating below the superior mesenteric artery
eGFR: Estimated glomerular filtration rate
EPF: Electronic patient file
GCP: Good clinical practice
GDPR: General data protection regulation
HIT: Heparin-induced thrombocytopenia
HMS: Hemostasis Management System
HR-ACT: High-range ACT cartridges
ICAC: Independent central adjudication committee
iMCQ: iMTA medical consumption questionnaire
iPCQ: iMTA productivity cost questionnaire
IU: International units
NCAP: Non-cardiac arterial procedures
RCT: Randomised controlled trial
RIFLE: Risk, injury, failure, loss, end stage renal disease; criteria for classifying the severity of acute kidney injury
SAE: Serious adverse event
SIV: Site initiation visit
SPC: Summary of product characteristics
SUSAR: Suspected unexpected serious adverse reaction
TEC: Thrombo-embolic complications
TIA: Transient ischemic attack
WMO: Medical research involving human subjects act
