# Supplementary material for: ACTION-1: study protocol for a randomised controlled trial on ACT-guided heparinization during open abdominal aortic aneurysm repair
Source: Trials. 2021 Sep 19;22:639. doi: 10.1186/s13063-021-05552-7 (PMC8449992; doi:10.1186/s13063-021-05552-7)
Supplement: Supplementary file 1 — Additional file 1:. Ethical approval document, official English summary included. [file 13063_2021_5552_MOESM1_ESM.pdf]

dr. A.M. Wiersema
staflid Heelkunde, WFG (kamer G084)
Maelsonstraat 3
1624 NP HOORN

Medisch Ethische Toetsingscommissie VUmc
Van der Boechorststraat 7, kamer H-443
Postbus 7057
1007 MB Amsterdam
020 444 5585
www.vumc.nl/METc

Datum:	Amsterdam, 21 februari 2020
Ons kenmerk:	2019.732 - NL66759.029.19
Betreft:	Positief oordeel geneesmiddelenonderzoek

Geachte heer Wiersema,

De Medisch Ethische Toetsingscommissie VU medisch centrum (bevoegd tot oordelen op grond van art. 2.2.a WMO) **oordeelt positief over de uitvoering van het onderzoek** met titel:

ACTION-1: ACT guided heparinization during open abdominal aortic aneurysm repair, a randomised trial.

Verrichter: Dijklander ziekenhuis (Hoorn en Purmerend)
Type onderzoek: Observationeel onderzoek (zonder invasieve metingen)
Subtype onderzoek: Onderzoek met een geneesmiddel

De goedkeuring van het protocol is gebaseerd op de documenten die in bijlage 1 zijn opgenomen.

Wij wijzen u erop dat **alle** onderzoekers die betrokken zijn bij de uitvoering van de studie (ook de onderzoekers in de deelnemende instellingen) op de hoogte moeten zijn van de laatste aanpassingen in het onderzoeksprotocol en eventuele appendices. Hiervan moet schriftelijk bewijs aanwezig zijn bij de onderzoeksdocumentatie.

- **Geen bezwaar Bevoegde Instantie (BI)**
  Het onderzoek mag pas van start gaan wanneer u van de BI (CCMO) een schriftelijke verklaring van geen bezwaar heeft ontvangen.
- **Toestemming raad van bestuur VUmc**
  Volledigheidshalve maken wij u erop attent dat het onderzoek pas mag worden uitgevoerd nadat u schriftelijk toestemming heeft gekregen van de raad van bestuur van VUmc.

AMC en VUmc werken samen in Amsterdam UMC

VUmc
De Boelelaan 1117
1081 HV Amsterdam
Postbus 7057
1007 MB Amsterdam
T +31(0)20 444 4444
www.vumc.nl

Deelnemende centra
De commissie heeft de in bijlage 1 vermelde onderzoeksverklaring(en) bekeken. Zij heeft geconstateerd dat is voldaan aan de voorwaarden in artikel 3 onderdeel e en k van de WMO.

De goedkeuring betreft de uitvoering in:

Onderzoekscentra	Lokale hoofdonderzoeker
Academisch Medisch Centrum	M.J.W. Koelemay
VU medisch centrum	J.D. Blankensteijn
Dijklander Ziekenhuis	V. Jongkind
Rijnstate ziekenhuis (loc. Arnhem)	M.P.J. Reijnen
Isala Klinieken	M. Pierie
Medisch Spectrum Twente	E. Willigendael
Alrijne Ziekenhuis locatie Leiderdorp	R. Hoencamp
Maasstad Ziekenhuis Rotterdam	B. Fioole
Leids Universitair Medisch Centrum	J. van Schaik
Amphia ziekenhuis	L. van der Laan
Elisabeth-TweeSteden Ziekenhuis	J. Heyligers
Groene Hart Ziekenhuis	P.M. Schlejen
Catharina ziekenhuis	J.A.W. Teijink
St Antonius ziekenhuis (Nieuwegein)	R.H.J. Kropman

Het bestuur of directie van deze instelling(en) dient toestemming te geven voor de uitvoering van het onderzoek in de eigen instelling. Pas na het verkrijgen van deze toestemming kan het onderzoek in het desbetreffende centrum starten.

Indien het onderzoek ook in een of meerdere Nederlandse instellingen dan bovengenoemd zal worden uitgevoerd, dient de coördinator van het onderzoek hiervoor een onderzoeksverklaring in te dienen, op grond waarvan de METc VUmc een nader oordeel zal uitspreken over de participatie van die instellingen.

Vergadering en documenten
Op 18/12/2019 is het onderzoeksdossier compleet verklaard en bij de METc VUmc in behandeling genomen. Het onderzoeksdossier - gebaseerd op de documenten die in bijlage 1 zijn vermeld - is besproken in de vergadering van 09/01/2020. De samenstelling van de commissieleden is in bijlage 2 opgenomen.

Motivering
De commissie is van oordeel dat het onderzoek voldoet aan het bepaalde in de van toepassing zijnde wet- en regelgeving, met name de WMO en, voor zover relevant, het ICH/GCP richtsnoer.

AMC en VUmc werken samen in Amsterdam UMC

VUmc
De Boelelaan 1117
1081 HV Amsterdam
Postbus 7057
1007 MB Amsterdam
T +31(0)20 444 4444
www.vumc.nl

Naar de mening van de commissie betreft het ingediende onderzoek een geneesmiddelenonderzoek zoals bedoeld in artikel 1, eerste lid onder n, van de WMO. Tevens is artikel 13a van de WMO van toepassing en is voldaan aan alle in artikel 13d van de WMO genoemde aanvullende voorwaarden.

De commissie is van oordeel dat het onderzoeksprotocol in een toestemmingsprocedure voorziet die overeenstemt met artikel 6, eerste lid, van de WMO.

De commissie is van mening dat is voldaan aan de voorwaarden in artikel 6, vijfde t/m negende lid, van de WMO. De proefpersonen (en/of degenen die mede/in hun plaats bevoegd zijn tot het geven van toestemming voor deelname aan het onderzoek) worden op gepaste, volledige en begrijpelijke wijze schriftelijk over het onderzoek geïnformeerd.

Verzekeringen
De METc VUmc heeft vastgesteld dat op correcte wijze uitvoering is gegeven aan de verzekeringsplicht zoals bepaald in artikel 7, eerste lid, van de WMO, zoals uitgewerkt in het *Besluit verplichte verzekering bij medisch-wetenschappelijk onderzoek met mensen 2015* (Besluit van 24 november 2014). Naar het oordeel van de commissie gaat het onderzoek gepaard met risico.

Het onderzoek valt onder de proefpersonenverzekering van Centramed.

De commissie heeft geconstateerd dat een aansprakelijkheidsverzekering is afgesloten zoals bepaald in artikel 7, negende lid, van de WMO.

Voorwaarden en verplichtingen
De METc VUmc wijst u op de voorwaarden en verplichtingen die in bijlage 3 zijn vermeld. De commissie heeft de bevoegdheid haar positieve oordeel in te trekken als vaststaat dat de uitvoering van het onderzoek ernstig tekort schiet.

Het voorliggend oordeel verliest zijn geldigheid indien de start van het onderzoek (inclusie van de eerste proefpersoon) niet binnen twee jaar nadat dit besluit is genomen heeft plaatsgevonden.

Administratief beroep
Tegen dit besluit kan een belanghebbende op grond van artikel 23 WMO binnen zes weken na de dag waarop het besluit is bekend gemaakt, administratief beroep instellen bij de Centrale Commissie Mensgebonden Onderzoek (CCMO). Het beroepschrift dient u te adresseren aan: CCMO, Postbus 16302, 2500 BH Den Haag.

Met vriendelijke groet,
namens de Medisch Ethische Toetsingscommissie,

i.o. D. Schouten   D. Schouten

prof. dr. J.A.M. van der Post, voorzitter   Mevr. D.I. Schouten, MSc, secretaris

AMC en VUmc werken samen in Amsterdam UMC

VUmc
De Boelelaan 1117
1081 HV Amsterdam

Postbus 7057
1007 MB Amsterdam

T +31(0)20 444 4444
www.vumc.nl

Bijlage 1: Documentenlijst
Bijlage 2: Samenstelling METc VUmc
Bijlage 3: Voorwaarden en verplichtingen
Bijlage 4: IRB approval

c.c.: Centrale Commissie Mensgebonden Onderzoek te Den Haag (CCMO) - *digitaal uploaden*
c.c.: L.C. Roosendaal / l.c.roosendaal@westfriesgasthuis.nl
c.c.: S. van Rossum / s.vanrossum@westfriesgasthuis.nl

AMC en VUmc werken samen in Amsterdam UMC

VUmc
De Boelelaan 1117
1081 HV Amsterdam

Postbus 7057
1007 MB Amsterdam

T +31(0)20 444 4444
www.vumc.nl

Bijlage 1: Documentenlijst

Sectie	Onderwerp	Versie
A1	aanbiedingsbrief	lijst met aanwezige documenten
A1	aanbiedingsbrief	METc VUmc d.d. 10-12-2019
A1	aanbiedingsbrief	RvB VUmc d.d. 17-1-2020
A1	commentaar METc	d.d. 10-1-2020
A1	commentaar METc	d.d. 6-2-2020
A1	commentaar METc	PIF d.d. 10-1-2020
A1	reactie op commentaar METc	d.d. 10-2-2020
A1	reactie op commentaar METc	d.d. 20-1-2020
A2	machtiging van de verrichter	RvB Dijklander Ziekenhuis t.b.v. A.M. Wiersema d.d. 20-5-2019
A3	ontvangstbewijs EudraCT-nummer	d.d. 29-8-2019
B1	ABR-formulier	versie 10 d.d. 10-2-2020
B24	risicoclassificatie	versie 2.0 d.d. 5-10-2019 (matig)
B3	EudraCT aanvraagformulier	getekend d.d. 10-2-2020
C1	onderzoeksprotocol	versie 10 d.d. 8-2-2020
C1	onderzoeksprotocol	versie 10 d.d. 8-2-2020 (TC)
D21	lijst relevante trials	Heparine versie 001 d.d. 5-10-2019
D21	SPC	Heparine versie 001 d.d. 12-10-2019
D21	SPC	Protaminehydrochloride d.d. 3-1-2019
D4	vergunningen/verklaringen	CASTOR beveiligingsverklaring
D4	vergunningen/verklaringen	CASTOR ISO 27001 d.d. 19-12-2017
D4	vergunningen/verklaringen	CASTOR NEN 7510 d.d. 19-12-2017
E12	informatiebrief incl. toestemmingsverklaring	versie 4 d.d. 8-2-2020
E12	informatiebrief incl. toestemmingsverklaring	versie 4 d.d. 8-2-2020 (TC)
F1	vragenlijst	CRF versie 445.81 d.d. 21-1-2020
F1	vragenlijst	eCRF versie 445.81 d.d. 21-1-2020
F1	vragenlijst	EQ-5D-5L versie 2019.002 d.d. 19-11-2019
F1	vragenlijst	iMTA MCQ versie 2020.003 d.d. 17-1-2020
F1	vragenlijst	iMTA MCQ versie 2020.003 d.d. 17-1-2020 (TC)
F1	vragenlijst	iMTA PCQ versie 4 d.d. 6-2-2020
F1	vragenlijst	iMTA PCQ versie 4 d.d. 6-2-2020 (TC)
G1	WMO proefpersonenverzekering	Centramed t.b.v. Dijklander Ziekenhuis d.d. januari 2020
G2	aansprakelijkheidsverzekering	Centramed t.b.v. AMC d.d. januari 2020
G2	aansprakelijkheidsverzekering	Centramed t.b.v. Catharina Ziekenhuis d.d. januari 2019
G2	aansprakelijkheidsverzekering	Centramed t.b.v. Groene Hart Ziekenhuis d.d. januari 2018
G2	aansprakelijkheidsverzekering	Centramed t.b.v. LUMC d.d. januari 2019
G2	aansprakelijkheidsverzekering	Centramed t.b.v. Sint Antonius Ziekenhuis d.d. januari 2019
G2	aansprakelijkheidsverzekering	Centramed t.b.v. VUmc d.d. januari 2019
G2	aansprakelijkheidsverzekering	MediRisk t.b.v. Alrijne Ziekenhuis d.d. 20-11-2018

AMC en VUmc werken samen in Amsterdam UMC

VUmc
De Boelelaan 1117
1081 HV Amsterdam
Postbus 7057
1007 MB Amsterdam
T +31(0)20 444 4444
www.vumc.nl

G2	aansprakelijkheidsverzekering	MediRisk t.b.v. Amphia Ziekenhuis d.d. 20-11-2018
G2	aansprakelijkheidsverzekering	MediRisk t.b.v. Elisabeth-TweeSteden Ziekenhuis d.d. 20-11-2018
G2	aansprakelijkheidsverzekering	MediRisk t.b.v. Maasstad Ziekenhuis d.d. 20-11-2018
G2	aansprakelijkheidsverzekering	MediRisk t.b.v. Medisch Spectrum Twente d.d. 20-11-2018
G2	aansprakelijkheidsverzekering	MediRisk t.b.v. Rijnstate Ziekenhuis d.d. 20-11-2018
G2	aansprakelijkheidsverzekering	Van Lanschot Chabot t.b.v. Isala Klinieken d.d. 4-3-2019
H1	CV onafhankelijk deskundige	M.H.J. Verhofstad d.d. 9-12-2019
I1	lijst deelnemende centra	versie 002 d.d. 11-8-2019
I2	onderzoeksverklaring	Alrijne Zorggroep t.b.v. R. Hoencamp d.d. 6-3-2019
I2	onderzoeksverklaring	AMC t.b.v. M.J.W. Koelemay d.d. 1-10-2019
I2	onderzoeksverklaring	Catherina Ziekenhuis t.b.v. J.A.W. Teijink d.d. 4-9-2019
I2	onderzoeksverklaring	Dijklander t.b.v. V. Jongkind d.d. 5-410-2019
I2	onderzoeksverklaring	Elisabeth-TweeSteden t.b.v. J.M.M. Heyligers d.d. 19-9-2019
I2	onderzoeksverklaring	Groene Hart t.b.v. P.M. Schlejen d.d. 11-9-2019
I2	onderzoeksverklaring	Isala t.b.v. M. Pierie d.d. 6-9-2019
I2	onderzoeksverklaring	LUMC t.b.v. J. van Schaik d.d. 10-9-2019
I2	onderzoeksverklaring	Maasstad t.b.v. B. Fioole d.d. 5-9-2019
I2	onderzoeksverklaring	Medisch Spectrum Twente t.b.v. E. Willigendael d.d. 4-9-2019
I2	onderzoeksverklaring	Rijnstate t.b.v. M.M.P.J. Reijnen d.d. 29-8-2019
I2	onderzoeksverklaring	Sint Antonius t.b.v. R.H.J. Kropman 10-10-2019
I2	onderzoeksverklaring	Stichting Amphia t.b.v. L. van der Laan d.d. 30-8-2019
I2	onderzoeksverklaring	VUmc t.b.v. J.D. Blankensteijn d.d. 5-9-2019
I31	CV hoofdonderzoeker	A.M. Wiersema (Dijklander Ziekenhuis) d.d. 12-10-2019
I32	CV uitvoerend onderzoeker	L. Roosendaal (Dijklander Ziekenhuis) d.d. 10-10-2019
I4	CV lokale onderzoeker	B. Fioole (Maasstadziekenhuis) d.d. 4-9-2019
I4	CV lokale onderzoeker	E. Willigendael (Medisch Spectrum Twente)
I4	CV lokale onderzoeker	J. Heyligers (Elisabeth-TweeSteden Ziekenhuis)
I4	CV lokale onderzoeker	J. van Schaik (LUMC)
I4	CV lokale onderzoeker	J.A.W. Teijink (Catharina Ziekenhuis) d.d. 25-2-2019
I4	CV lokale onderzoeker	J.D. Blankensteijn (VUmc) d.d. 5-9-2019
I4	CV lokale onderzoeker	L. van der Laan (Amphia Ziekenhuis)
I4	CV lokale onderzoeker	M. Pierie (Isala Klinieken)
I4	CV lokale onderzoeker	M.J.W. Koelemay (AMC)
I4	CV lokale onderzoeker	M.P.J. Reijnen (Rijnstate Ziekenhuis) d.d. 27-8-2019
I4	CV lokale onderzoeker	P.M. Schlejen (Groene Hart Ziekenhuis)
I4	CV lokale onderzoeker	R.H.J. Kropman (Sint Antonius Ziekenhuis)
I4	CV lokale onderzoeker	V. Jongkind (Dijklander Ziekenhuis) d.d. 18-8-2019
K1	beoordeling andere instantie	indiening marginale toets BI d.d.10-12-2019
K1	beoordeling andere instantie	ZonMw subsidieaanvraag d.d. 5-12-2019
K3	onderzoekscontract	CTA Dijklander Ziekenhuis, Rijnstate Ziekenhuis en M.M.P.J. Reijnen getekend d.d. 29-10-2019
K4	wetenschappelijke publicatie (sectie	Doganer et al. 2019

AMC en VUmc werken samen in Amsterdam UMC

VUmc
De Boelelaan 1117
1081 HV Amsterdam

Postbus 7057
1007 MB Amsterdam

T +31(0)20 444 4444
www.vumc.nl

	K)	
K5	DSMB_charter	versie 3.0 (TC)
K5	DSMB_charter	versie 3.0 d.d. 17-1-2020
K5	DSMB_samenstelling	d.d. 11-10-2019
K6	overig sectie K	governance en samenstelling commissies en projectgroepen versie 2 d.d. 15-10-2019
K6	overig sectie K	WMO/GCP certificaat Wiersema d.d. 20-1-2019

AMC en VUmc werken samen in Amsterdam UMC

VUmc
De Boelelaan 1117
1081 HV Amsterdam

Postbus 7057
1007 MB Amsterdam

T +31(0)20 444 4444
www.vumc.nl

Bijlage 2: Samenstelling METc VUmc

dhr. prof. dr. J.A.M. van der Post	voorzitter, gynaecoloog
dhr. dr. M.J.J. Finken	vice-voorzitter, kinderarts
dhr. dr. P.M. Bet	ziekenhuisapotheker-klinisch farmacoloog (plv.)
mevr. mr. L. Brakel	jurist
mevr. dr. M.A. Bremmer	psychiater
dhr. dr. J. Buter	internist-oncoloog
dhr. dr. B. Drukarch	arts-farmacoloog
dhr. dr. ir. Ing. Th.J.C. Faes	klinisch fysicus
dhr. dr. ing. M. Grimbergen	klinisch fysicus (plv.)
dhr. dr. E.G. Haarman	kinderarts
dhr. dr. A.W.J. Hoksbergen	chirurg (plv.)
dhr. dr. S. Idema	neurochirurg
dhr. dr. M.J.P.A. Janssens	medisch ethicus
dhr. prof. dr. M. Klein	neuropsycholoog
dhr. dr. M.D. Lagerweij	tandarts
mevr. dr. J.H. van der Lee	methodoloog (plv.)
mevr. dr. B.I. Lissenberg-Witte	methodoloog (plv.)
mevr. dr. H. van Luijn	medisch ethicus (plv.)
mevr. L. Muter	proefpersonenlid (plv.)
mevr. G. Nijman	proefpersonenlid
mevr. A. Rutte-Stiekema	stralingsdeskundige (plv.)
mevr. dr. C.B. Terwee	methodoloog (plv.)
dhr. dr. ir. P. van de Ven	methodoloog
dhr. ing. S.W. Vianen	stralingsdeskundige
mevr. prof. dr. van der Vies	biochemicus
mevr. mr. M. Wildenbeest	jurist (plv.)
dhr. drs. A.J. Wilhelm	ziekenhuisapotheker-klinisch farmacoloog

AMC en VUmc werken samen in Amsterdam UMC

VUmc
De Boelelaan 1117
1081 HV Amsterdam

Postbus 7057
1007 MB Amsterdam

T +31(0)20 444 4444
www.vumc.nl

Bijlage 3: Voorwaarden en verplichtingen

Geldigheid oordeel
Het positieve oordeel verliest zijn geldigheid als de inclusie van de eerste proefpersoon niet heeft plaatsgevonden binnen twee jaar nadat dit besluit is genomen.

Onderzoekscontract
Een getekend exemplaar van de goedgekeurde versie van het onderzoekscontract moet vóórdat de studie zal starten ter kennisgeving bij de toetsingscommissie worden ingediend.

Geldigheid verzekering
In het geval het verzekeringscertificaat tijdens de voortgang van het onderzoek zijn geldigheid verliest, dient aan de METc VUmc tijdig een afschrift van een nieuw geldig certificaat te worden toegestuurd.

Amendementen
Amendementen dienen ter beoordeling aan de METc VUmc te worden voorgelegd.

Startdatum onderzoek
De METc VUmc dient op de hoogte te worden gesteld van de definitieve startdatum van het onderzoek. Dat is de datum waarop de inclusie van de eerste proefpersoon plaatsvindt.

Voortgangsrapportage
Eén jaar na datum van het oordeel, en ieder jaar daaropvolgend, dient de METC op de hoogte te worden gebracht van de voortgang van de studie middels het formulier Voortgangsrapportage.

Veiligheidsrapportage
Eén jaar na datum van het oordeel, en ieder jaar daaropvolgend, dient bij geneesmiddelenonderzoek aan de METC een veiligheidsrapportage te worden gestuurd. De veiligheidsrapportage kan gecombineerd worden met de jaarlijkse voortgangsrapportage.

Melding SAE's
SAE's dienen aan de METc VUmc te worden gemeld.

Melding SUSARs
SUSARs dienen aan de METc VUmc te worden gemeld.

Advies DSMB
Indien een advies van de DSMB niet volledig wordt opgevolgd, dient de METc VUmc het advies met toelichting over het niet (volledig) opvolgen van het advies te ontvangen en toestemming te geven voor voortzetting van het onderzoek.

Melding (voortijdige) beëindiging en opschorting
(Voortijdige) beëindiging en opschorting van het onderzoek dient, met redenen omkleed, te worden gemeld aan de METc VUmc.

AMC en VUmc werken samen in Amsterdam UMC

VUmc
De Boelelaan 1117
1081 HV Amsterdam
Postbus 7057
1007 MB Amsterdam
T +31(0)20 444 4444
www.vumc.nl

Eindrapportage
De METc VUmc dient op de hoogte te worden gebracht van de resultaten van het onderzoek middels een eindrapport.

Termijnen en overige uitleg ten aanzien van de indiening van de verschillende documenten aan de METc VUmc *vindt u op de website (www.vumc.nl/afdelingen/METc/).*

AMC en VUmc werken samen in Amsterdam UMC

VUmc
De Boelelaan 1117
1081 HV Amsterdam

Postbus 7057
1007 MB Amsterdam

T +31(0)20 444 4444
www.vumc.nl

Bijlage 4: IRB approval

dr. A.M. Wiersema
staflid Heelkunde, WFG (kamer G084)
Maelsonstraat 3
1624 NP HOORN

Medical Ethics Review Committee VUmc
Van der Boechorststraat 7, room H-443
P.O. box 7057
1007 MB Amsterdam
+31 (0)20 444 5585
www.vumc.nl/METc

Date:	Amsterdam, 21 February 2020
Our reference:	2019.732 - NL66759.029.19
Subject:	IRB approval multicenter study with medicinal product

Dear Mr. Wiersema,

The Institutional Review Board of VU University Medical Center (hereafter: METc VUmc) in Amsterdam, competent to review in accordance with section 2.1a of the Medical Research Involving Human Subjects Act (WMO), **gives approval for the study** with the title:

ACTION-1: ACT guided heparinization during open abdominal aortic aneurysm repair, a randomised trial.

Initiator: Dijklander ziekenhuis (Hoorn en Purmerend)
Type of research: Observation research
Subtype: Research with a medicinal product

Participating centers
The approval concerns execution of the research protocol to be performed in the Netherlands in the following center(s):

Research center	Local Principal Investigator
Academisch Medisch Centrum	M.J.W. Koelemay
VU medisch centrum	J.D. Blankensteijn
Dijklander Ziekenhuis	V. Jongkind
Rijnstate ziekenhuis (loc. Arnhem)	M.P.J. Reijnen
Isala Klinieken	M. Pierie
Medisch Spectrum Twente	E. Willigendael
Alrijne Ziekenhuis locatie Leiderdorp	R. Hoencamp
Maasstad Ziekenhuis Rotterdam	B. Fioole

Leids Universitair Medisch Centrum	J. van Schaik
Amphia ziekenhuis	L. van der Laan
Elisabeth-TweeSteden Ziekenhuis	J. Heyligers
Groene Hart Ziekenhuis	P.M. Schlejen
Catharina ziekenhuis	J.A.W. Teijink
St Antonius ziekenhuis (Nieuwegein)	R.H.J. Kropman

Committee meetings and documents

The research documents were discussed during the plenary meeting which took place on 01/09/2020. The approval is based on the following documents:

- the research protocol d.d. 02/08/2020 version 10
- written information and consent statement to be used d.d. 8-2-2020 version 4

With regard to the other documents approved, we refer to appendix 1 (bijlage 1) of the original decision in Dutch.

Motivation

The committee is of the opinion that the study is in compliance with all current legislation, in particular the WMO and, if relevant, the ICH/GCP.

The METc VUmc is registered with the US Office for Human Research Protections (OHRP) as IRB00002991. The FWA number assigned to VU University Medical Center is FWA00017598.

AMC en VUmc werken samen in Amsterdam UMC

VUmc
De Boelelaan 1117
1081 HV Amsterdam

Postbus 7057
1007 MB Amsterdam

T +31(0)20 444 4444
www.vumc.nl
