# Supplementary material for: ACTION-1: study protocol for a randomised controlled trial on ACT-guided heparinization during open abdominal aortic aneurysm repair
Source: Trials. 2021 Sep 19;22:639. doi: 10.1186/s13063-021-05552-7 (PMC8449992; doi:10.1186/s13063-021-05552-7)
Supplement: Supplementary file 2 — Additional file 2:. Original funding documentation. [file 13063_2021_5552_MOESM2_ESM.pdf]

Nederlandse organisatie voor gezondheidsonderzoek en zorginnovatie

Laan van Nieuw Oost-Indië 334
2593 CE Den Haag
Postbus 93245
2509 AE Den Haag
Telefoon 070 349 51 11
Fax 070 349 51 00
www.zonmw.nl
info@zonmw.nl

Dijklander Ziekenhuis
De heer Prof. dr. H.A. Keuzenkamp
Postbus 600
1620 AR HOORN NH

Dossiernummer
80-84800-98-43019
Ons kenmerk
2019/30247/ZONMW

Datum
5 december 2019

Contactpersoon
Marjo Tieleman
Telefoon 070 349 5464
geneesmiddelen@zonmw.nl

Onderwerp
Honorering van uw subsidieaanvraag, projectnummer 848043004

Geachte heer Keuzenkamp,

Met genoegen laat ik u weten dat de commissie Goed Gebruik Geneesmiddelen een positief oordeel heeft over uw aanvraag 80-84800-98-43019 met de titel '*ACTION-1: ACT guided heparinization during open abdominal aortic aneurysm repair, a randomised trial*'. Dat betekent dat ZonMw u de subsidie zal toekennen. In deze brief leest u wat u moet doen voordat uw project van start kan gaan.

Beoordeling
Voor de beoordeling verwijs ik u graag naar de brief met het voorgenomen besluit van 22 februari 2019. De commissie is van mening dat u middels uw schriftelijke reacties en aangeleverde documenten van 7 mei en 2 juli 2019 aan de aanvullende voorwaarden heeft voldaan. De commissie heeft de volgende opmerkingen naar aanleiding van uw reacties:

- Zoals door u aangegeven zijn de definities van TEC gebaseerd op de huidige (inter)nationale publicaties/normeringen voor open herstel van een AAA. Hierbij gelden twee standaardpublicaties en de DSAAA registratie als de norm. In het protocol dient dit duidelijk te worden aangegeven met verwijzing waar dit op gebaseerd is; voor toekomstige publicaties is dit essentieel.
- De gegevens die ten grondslag liggen aan deze definities worden gehaald uit het SOP OK verslag en EPD, die uiteindelijk hun weerslag vinden in het CRF dat een periode van 30 dagen betreft en retrospectief wordt ingevuld. De commissie is van mening dat dit kwetsbaar is, ook aangezien het EPD door meerdere personen wordt ingevoerd. In het protocol dient duidelijk de vertaalslag van de diverse onderdelen van het CRF naar de eindpunten te worden vastgelegd. Hetzelfde geldt voor veneuze complicaties en bloedingscomplicaties.
- Voor de bloedingscomplicaties wordt de E-CABG classificatie gebruikt. In het protocol dient dit duidelijk te worden aangegeven met verwijzing waar dit op gebaseerd is.

U wordt verzocht uiterlijk bij de eerste milestone (zie hieronder) het definitieve protocol aan te leveren, waarbij wordt aangegeven welke wijzigingen er zijn ten opzichte van het in de subsidieaanvraag aangeleverde protocol.

Onze hoofdopdrachtgevers zijn VWS en NWO

Financiering

Hoogte subsidiebedrag

De financiële bijdrage van ZonMw voor uw project bedraagt, op basis van uw ingediende begroting d.d. 7 mei 2019, maximaal € 1.635.600,- voor de duur van maximaal 60 maanden (januari 2020 tot en met december 2024). Dit bedrag is inclusief eventueel verschuldigde BTW.
Er is een correctie toegepast voor de opgevoerde benchfee. Deze kan niet worden opgevoerd als niet-academisch medisch centrum. De correctie bedraagt -/- € 10.000,-. Tevens is een correctie toegepast voor de opgevoerde BTW component voor de researchnurses. Deze kan niet worden opgevoerd als zodanig, aangezien researchnurses als eigen personeel staan vermeld.
De correctie bedraagt -/- € 52.500,-.
Wij verzoeken u om duidelijke specificaties van de verschillende posten bij de financiële eindafrekening bij het einde van het project, zowel voor de totale kosten als de bijdragen van eigen instelling en derden. De startdatum van uw project is 1 januari 2020. Omdat u al werkzaamheden heeft verricht aan uw project, accepteert ZonMw dat u kosten declareert in de periode van 1 oktober 2019 tot en met 31 december 2019.

Bevoorschottingen

ZonMw zal een bevoorschotting uit betalen op basis van het behalen van onderstaande milestones tijdens uw project. U dient zelf bij ZonMw kenbaar te maken wanneer de volgende milestone is behaald. Vervolgens zal ZonMw overgaan tot uitbetaling hiervan.

Milestone	Bevoor-schotting	Bedrag	Indicatiedatum
Start of project	15%	€ 245.340	januari 2020
1. METC and CCMO approval	10%	€ 163.560	april 2020
2. First Subject In (first centre)	10%	€ 163.560	mei 2020
3. First Subject In (last centre)	5%	€ 81.780	december 2020
4. Interim-analyses (after primairy endpoint 100 patients)	5%	€ 81.780	maart 2021
5. 20% of inclusion completed (150 patients)	10%	€ 163.560	juni 2021
6. Interim-analyses (after primairy endpoint 200 patients)	5%	€ 81.780	september 2021
7. 50% of inclusion completed (375 patients)	10%	€ 163.560	juni 2022
8. Interim-analyses (after primairy endpoint 500 patients)	5%	€ 81.780	januari 2023
9. 80% of inclusion completed (600 patients)	10%	€ 163.560	juni 2023
10. Last Subject In	5%	€ 81.780	februari 2024
11. Statistical Analyses	5%	€ 81.780	oktober 2024
Clinical Study Report én Goedgekeurd eindverslag inclusief financiële afrekening	5%	€ 81.780	december 2024

Bij het melden dat een milestone is behaald en het verzoek aan ZonMw om de volgende bevoorschotting uit te betalen, dient u tevens aan te geven of de financiering gedekt is gezien het verloop van werkelijk gemaakte kosten.
U dient tijdig (en niet gekoppeld aan een milestone) aan te geven bij ZonMw als de dekking in gevaar komt voor het resultaat van het project.

De commissie zal op onderstaande milestones een uitspraak doen over de voortgang van de studie:

- Milestone 4; interim-analyses (after primary endpoint 100 patients)
- Milestone 5; 20% of inclusion completed (150 patients)
- Milestone 6; interim-analyses (after primary endpoint 200 patients)
- Milestone 7; 50% of inclusion completed (375 patients)
- Milestone 8; interim-analyses (after primary endpoint 500 patients)

Op basis van bovenstaande momenten zal ZonMw de subsidie continueren, dan wel beëindigen. Indien er aanleiding toe is kan de commissie besluiten tot het inplannen van extra go/no go momenten.

Subsidievoorwaarden
Zoals u weet zijn aan de financiering voorwaarden verbonden. Deze subsidievoorwaarden kunt u downloaden via de website van ZonMw: www.zonmw.nl/subsidievoorwaarden
Ik wil u erop wijzen dat ZonMw pas een voorschot uitkeert als aan alle eisen voor het uitvoeren van het onderzoek is voldaan. Ik raad u dan ook aan eventuele procedures hiervoor tijdig te starten. Denkt u bijvoorbeeld aan een positief oordeel van een erkende medisch-ethische toetsingcommissie (METC), de Centrale Commissie Mensgebonden Onderzoek (CCMO), of een vergunning krachtens de Wet op het Bevolkingsonderzoek (WBO). Als u niet zeker weet of uw project dergelijke verklaringen of vergunningen nodig heeft, kunt u dit nagaan bij de betreffende instanties. Bij gerandomiseerd onderzoek met patiënten dient u de gegevens van de studie aan te melden bij het Nederlands Trial Register (NTR) (www.trialregister.nl) en op www.clinicaltrials.gov.

Integriteit
Artikel 2, lid 3 van de Subsidiebepalingen van ZonMw impliceert dat de nationaal en internationaal aanvaarde normen van wetenschappelijk handelen worden nageleefd zoals neergelegd in de Nederlandse gedragscode wetenschappelijke integriteit (2018), dan wel vergelijkbare codes voor niet-universitaire instellingen. In geval van (mogelijke) schending van voornoemde normen bij een door ZonMw gefinancierd project, dient ZonMw hiervan onverwijld op de hoogte te worden gesteld en dienen alle ter zake relevante documenten aan ZonMw te worden overlegd.

ZonMw bepaalt dat de bijlage Akkoord bekostiging wetenschappelijk onderzoek 2008 en het addendum, conform artikel 7 van het akkoord niet integraal van toepassing zijn op deze subsidie. Deze worden zoveel mogelijk analoog toegepast voor zover het akkoord of het addendum niet strijdig zijn met de Algemene subsidiebepalingen van ZonMw. De Algemene subsidiebepalingen van ZonMw zijn te allen tijde leidend. Zo zal ZonMw bijvoorbeeld altijd afrekenen op basis van werkelijke kosten.

Wat moet u doen?
Belangrijk: schriftelijke bevestiging
ZonMw kan u een voorschot tot het moment van de eerste milestone (METC and CCMO approval) verstrekken. Dit is echter pas mogelijk als u heeft ingestemd met de subsidievoorwaarden en het project daadwerkelijk gestart is. Wilt u daarom **uiterlijk 31 december 2019** schriftelijk onderstaande informatie doorgeven aan ZonMw? Hiervoor kunt u gebruikmaken van het bijgevoegde meldingsformulier:

- uw instemming met de voorwaarden die van toepassing zijn op de toekenning van de financiële bijdrage;
- de startdatum van **1 januari 2020** van uw project;
- de bank- en referentiegegevens voor de betalingen van de subsidie;
- ten aanzien van de goedkeuring van de METC:
    - Als de verklaring(en) noodzakelijk is voor de start van het project, stuurt u de verklaring voor de start van het project aan ZonMw.
    - In het geval de verklaring(en) pas later in het project vereist is, geeft u aan wanneer de verklaring(en) nodig is. Dit is maximaal één jaar na de start van het project. ZonMw keert dan een voorschot uit tot de eerste milestone (METC and CCMO approval). Verdere voorschotten kan ZonMw alleen betalen als een kopie van de verklaring(en) is ontvangen en de milestones zijn behaald.

ZonMw accepteert de sponsorovereenkomst met Medtronic (A1531759 / ERP-2018-11605, ondertekent door Medtronic op 24 oktober 2019).

ZonMw accepteert de final draft Consortium Agreement (ingediend op 11 oktober 2018). ZonMw ontvangt binnen 6 maanden na start van het project een kopie van de door alle genoemde partijen ondertekende Consortium Agreement.

Indien niet beschreven in het onderzoeksprotocol dient vóór Database Lock een Statistical Analyses Plan opgestuurd te worden.

Ik wijs u erop dat het project **uiterlijk zes maanden** na dagtekening van deze brief moet beginnen. Gaat het project later van start, dan vervalt de honorering van uw aanvraag. Hiervan kan alleen in zeer bijzondere gevallen worden afgeweken.

Publiekssamenvatting
ZonMw publiceert alle gehonoreerde projecten op haar website met een leesbare Nederlandse samenvatting. Deze is bedoeld voor een breed geïnteresseerd publiek met verschillende achtergronden, op taalniveau eind VWO. Zie de schrijfwijzer op http://www.zonmw.nl/nl/over-zonmw/logo-huisstijl. Wij verzoeken u deze Nederlandse publiekssamenvatting zo spoedig mogelijk, maar tenminste binnen vier weken na dagtekening van deze brief aan te leveren. Hiervoor kunt u in ProjectNet het tekstvak Publiekssamenvatting gebruiken (maximaal 1000 karakters, inclusief spaties).

Voortgang
ZonMw wil graag op de hoogte blijven van de voortgang van uw project;

- Vanaf de start van de inclusie ontvangt ZonMw maandelijks een update van de actuele patiënteninclusie. ZonMw levert hiervoor het format aan (wordt separaat per email naar hoofdaanvrager gestuurd) en is gebaseerd op de door de hoofdaanvrager aangeleverde inclusieplanning (dd. 16 oktober 2019) waarin vanaf inclusiestart de actuele inclusie tevens wordt weergegeven. In geval de patiënteninclusie afwijkt ten opzichte van de aangeleverde inclusieplanning, kan ZonMw de frequentie van het aanleveren van updates van actuele inclusieplanning aanpassen.
- ZonMw werkt met een midterm voortgangsrapportage die u halverwege het project indient.
- Daarnaast ontvangt u van het programmasecretariaat na uw melding van het behalen van milestones 4, 5, 6, 7 en 8 het verzoek een korte rapportage over de stand van zaken van uw project in te dienen.
- De commissie monitort de voortgang van het project. Hiervoor worden gedurende de looptijd enkele bijeenkomsten georganiseerd waaraan uw deelname verplicht is.
- Tenslotte bent u verplicht tussentijdse wijzigingen te melden aan ZonMw. Pas na goedkeuring door ZonMw zijn de wijzigingen toegestaan.

Kennisbenutting
Resultaten van het project kunnen toepassing vinden in de praktijk, maar ook een rol spelen bij het maken van beleid, een volgende stap vormen in een wetenschappelijke carrière of de basis vormen voor een nieuw project. Om aan te geven wat er met de resultaten gebeurt, stellen wij u in voortgangs- en eindverslag diverse vragen over verspreiding- en implementatie. Ook dienen publicaties over en resultaten van het project tot vier jaar na afronding via ProjectNet aan ZonMw te worden aangeboden. Daarnaast bent u verplicht om ZonMw in deze periode te informeren over het gebruik van de resultaten.

ZonMw

Dossiernummer
80-84800-98-43019
Ons kenmerk
2019/30247/ZONMW

Datum
5 december 2019

Datamanagement
Op basis van artikel 20 van de Subsidiebepalingen van ZonMw moeten alle subsidieontvangers een datamanagementplan opstellen. Deze heeft u als bijlage bij de subsidieaanvraag ingediend. Gedurende uw project kunt u wijzigingen of aanvullingen doorvoeren en dient u de nieuwe versie aan ZonMw voor te leggen.
Publicatie van en toegang tot ruwe data van de studie dienen conform de geldende wet- en regelgeving en de richtlijnen van het European Medicines Agency (EMA) en de Centrale Commissie Mensgebonden Onderzoek (CCMO) uitgevoerd. Het beleid van uw instelling ten aanzien van het vrijgeven van de ruwe data en data listings die in het kader van deze studie worden gecreëerd, en op basis van welke criteria ze worden vrijgegeven dient in overeenstemming te zijn met de Algemene Subsidiebepalingen ZonMw en de specifieke bepaling in de oproeptekst van Grote Trials 'Publicatie van en toegang tot ruwe data van de studie worden conform de geldende wet- en regelgeving en de richtlijnen van het European Medicines Agency (EMA) en de Centrale Commissie Mensgebonden Onderzoek (CCMO) uitgevoerd'. Indien dit beleid niet in overeenstemming is, kan ZonMw gebruik maken van artikel 19.4 uit de Algemene Subsidiebepalingen ZonMw door het stellen van nadere voorwaarden aan de auteursrechten en de extractierechten op databanken die in het kader van het project worden gecreëerd.

Mocht u nog vragen hebben over deze brief, neemt u dan gerust contact op met de medewerker die in het briefhoofd vermeld staat. Als u ontevreden bent over de wijze waarop ZonMw uw aanvraag heeft behandeld, kunt u dit signaleren of een klacht indienen (zie hieronder). Vermeld in uw communicatie met ZonMw altijd het projectnummer. Nu uw aanvraag gehonoreerd is, vervalt het oorspronkelijke nummer en geldt het **nieuwe projectnummer 848043004**.

Ik wil u nogmaals feliciteren met de honorering van uw subsidieaanvraag. Veel succes bij de uitvoering van uw project!

Met vriendelijke groet,
namens het bestuur,

Henk J. Smid
directeur

Bijlage(n)
Meldingsformulier start project (bestemd voor hoofdaanvrager/projectleider penvoerder)

Kopie
Drs. A.M. Wiersema MD

Tegen deze beschikking kunt u bezwaar maken. In dat geval stuurt u binnen zes weken na de dag waarop het besluit bekend is gemaakt een bezwaarschrift aan het bestuur van ZonMw, t.a.v. Commissie Bezwaarschriften ZonMw, Postbus 93 245, 2509 AE Den Haag. Meer informatie over bezwaar maken? Raadpleeg de website: www.zonmw.nl/signalerenklagenbezwaarmaken
