# Supplementary material for: ACTION-1: study protocol for a randomised controlled trial on ACT-guided heparinization during open abdominal aortic aneurysm repair
Source: Trials. 2021 Sep 19;22:639. doi: 10.1186/s13063-021-05552-7 (PMC8449992; doi:10.1186/s13063-021-05552-7)
Supplement: Supplementary file 3 — Additional file 3:. English translation of original funding documentation. [file 13063_2021_5552_MOESM3_ESM.docx]

Translation of final letter of approval from ZonMw date 05 Dec 2019:

With pleasure we let you know that the committee of the program of Goed Gebruik Geneesmiddelen (GGG) has a positive verdict on your application 80-84800-98-43019, titled *‘ACTION-1: ACT guided heparinization during open abdominal aortic aneurysm repair, a randomized trial’*. This means that ZonMw will grant you the subsidy. In this letter you will read what you should do before your project can start.

**Review**

For the final review I refer to our letter with the intended decision of 22 feb 2019. The committee has decided that you have met the extended demands by your letters dating 7 may 2019 and 2 July 2019. The committee has the following remarks after reviewing your comments:

- Definitions of TEC are based on (inter)national publications and scoring systems for open repair of AAA. Two publications of standards and DSAA registration are considered to be setting the norm. In the protocol this need to be explicitly depicted with literature reference; this is considered to be essential for future publications

- Data on which these definitions and scoring of TEC are based on SOP OR-report and EPF, that are gathered in the CRF, which is limited on 30 days and is retrospectively filled in. The committee thinks this might be vulnerable, also considering the fact that the EPF is filled in by many healthcare professionals. In the protocol it needs to be explicitly depicted how the subjects of CRF are scored to judge the primary and secondary endpoints. The same is applicable to venous complications and bleeding complications.

You are demanded to provide us with the definite protocol before the first milestone (see further), in which it should be highlighted which changes are made compared to previous protocol.

**Finance**

*Grant total*

Financial support from ZonMw for your project is, based on your budget dating 7 may 2019, 1.635.600,-- euros for the duration of 60 moths (January 2020 – December 2024). This amount is including possible VAT.

A correction was made for the included bench fee. This cannot be granted as a non-academic medical center. This correction is -/- 10.000 euros. Also, a correction was made for the VAT component for the research nurses. This cannot be granted as research nurses are considered to be personnel of center. These corrections amount the total of -/- 52.000 euros.

We ask you to provide us with detailed specification of the budget subjects at the end of the project, for both the total costs as the adjunctive amounts from your own hospital and third parties. Starting of your project is 1 January 2020. Because you have already performed activities directly involving your project, ZonMw accepts you to declare costs in the timeframe from 1 October until 31december 2019.

*Advance payments*

ZonMw wil provide you with advance payments based on the following milestones during your project. You will notify ZonMw when applicable milestones have been achieved. Thereafter ZonMw will see to payments.


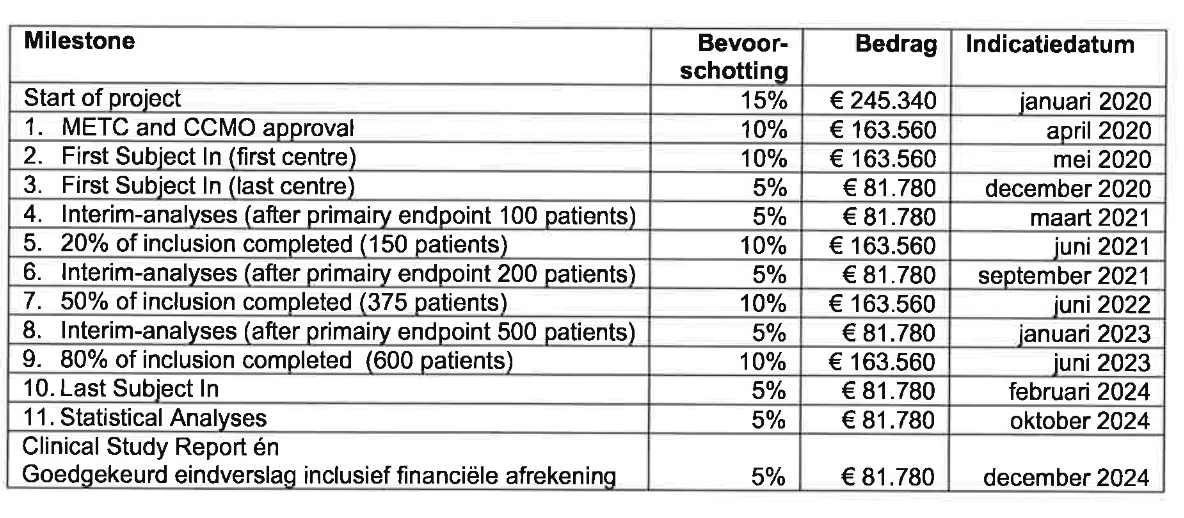


When notifying ZonMw of a reached milestone and the request for payment, we ask you to declare that the finacing is covering all made expenses. You have to notify ZonMw timely (and not solely at the point of a milestone, that coverage of your project is not jeopardized.

The committee of GGG will judge about continuation of your project during the following milestones:


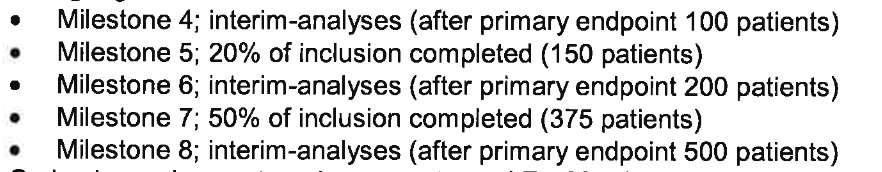


Based on the above depicted analyses ZonMw will continue the grant or stop it. If desired and applicable the committee can decide to implement extra go/no go moments.

*Subsidy conditions*

As known, there are conditions for financing the CTION-1 study. These conditions can be downloaded through the website of ZonMw: [www.zonmw.nl/subsidievoorwaarden](http://www.zonmw.nl/subsidievoorwaarden)

i would like to point out that ZonMw can only supply you with an advance payment when all conditions are met for executing your research. I advise you therefor to start eventual procedures as early as possible. For example, a positive advice of the Local Medical Ethics Committee (METC), the Central Committee on Human Research (CCMO), or a license for population research (WBO). If you are not sure whether your project needs such declarations, permissions or permits, you can contact the concerning agencies. For randomized trials with patients you need to register your study with the Dutch Trial Register (NTR) ([www.trialregister.nl](http://www.trialregister.nl)) and with [www.clinicaltrials.gov](http://www.clinicaltrials.gov)

*Integrity*

Article 2, section 3 of the subsidy conditions, implicates that national and international accepted standards of scientific research are being met, as depicted in the Dutch gedragscode wetenschappelijke integrity (2018), or comparable codes for non-academic institutions. In case of (possible) violations of these codes during a ZonMw financed project, ZonMw should be notified immediately and all applying documents are to be sent to ZonMw.

ZonMw states that the adjunctive Agreement funding scientific research 2008 and the addendum, conform Article 7, are not integral applied to this subsidy. These are as possible analogue applied to the agreement as far as the addendum is not contrary with the General subsidy conditions from ZonMw. These General Subsidy Conditions of ZonMw are leading at all times. As an example, ZonMw will always pay on the basis of actual costs.

**What do you have to do?**

*Important: written confirmation*

ZonMw can provide you with a deposit until the payment of the first milestone (METC and CCMO approval). This can only be possible if you have agreed to our Subsidy conditions and the project is actually started. So please provide ZonMw with the following information. You can use the attached form:

- your approval of the conditions applicable on the award of the financial subsidy

- starting date of your project will be January- 2020

- your bank and references data for the actual payment of the subsidy

- considering METC approval:

- if a positive decision is demanded before start of the project, send the official letter of the METC to ZonMw

- in case this letter is needed later in the project you supply details of when this letter is necessary. This is maximally 1 year after start of the project. ZonMw will supply you with an advance payment before first milestone (METC and CCMO approval). Further advance payments can only be supplied by ZonMw if a copy of these approvals are received by ZonMw and the milestones are reached.

ZonMw accepts the sponsor contract with Medtronic (A1531759 / ERP-2018-11605, signed by Medtronic dd 24 oct 2019).

ZonMw accepts the final draft Consortium Agreement (submitted on 11 oct 2019). ZonMw will receive a signed copy of the Consortium Agreement within 6 months of the start of the project.

If not specified in the research protocol, a Statistical Analyses Plan needs to be sent to ZonMw before Database Lock.

I would like to inform you that the project has to start at the latest 6 months after date of this letter. If the project starts later, your grant approval will be withdrawn. Only in very special circumstances this can be differed from.

*Public summary*

ZonMw publish all honored projects on its website with a readable Dutch lay summary. This is intended for a broad audience with different levels, on late high school language level. See the writing instructions on htttp://www.zonmw.nl/nl/over-zonmw/logo-huisstijl. We ask you to send us this Dutch public summary as soon as possible, but at least within 4 weeks after date of this letter. In ProjectNet you can use the textbox Public summary (max 1000 characters, including spaces).

*Progress*

ZonMw wants to be kept informed on the progress of your project.

- From the start of the inclusion, ZonMw will receive a monthly update of actual patient inclusion. ZonMw will provide you with the format for this (will separately be sent by mail to PI) and this will be based on the planned inclusion sent by PI (16 oct 2019). In case this patient inclusion differs from the planned inclusion, ZonMw may adjust the update frequency of actual inclusion.
- ZonMw incorporates a midterm progress report which you submit at half way the project.
- Apart from the above you will receive a request from the program secretary at reaching milestone 4, 5, 6, 7 and 8, to provide a short update of the progress of your project.
- The commission of ZonMw monitors the progress of the project. During the project a number of meetings will be held in which your participation is mandatory.
- Finally, you are obliged to report interim substantial changes of the project to ZonMW. Only after a permission of ZonMw these changes are allowed.

*Knowledge utilization*

Results of the project can be implemented in daily practice, but also be of important value of making policy, be a next step in a scientific career or be the basis for a new project. To show what will happen with the results of the project, ZonMw will be asking questions in progress and final reports. Also publications on and the results of the project until 4 years after end of project must be submitted to ZonMw through Projectnet. Apart from this condition you are also obliged to inform ZonMw about the use of results in this timeframe (4 years).

*Data management*

Based on article 20 of the Subsidiebepalingen of ZonMw, all projects must draft a data management plan. You have submitted such a plan with your application. During your project you can implement changes or supplements, which have to be submitted to ZonMw creating a new version.

Publication of and access to raw data of the study have to compile to all applicable law and guidelines of the European Medicines Agency (EMA) and CCMO. The policy and rules of your local institution concerning sharing raw data and data listings of the project, have to conform to the Algemene Subsidiebepalingen van ZonMw and all specific demands of the Grote Trials “Publicatie van en toegang tot ruwe data van de studie worden conform de geldende wet- en regelgeving van het European Medicines Agency (EMA) en de Centrale Commissie Mensgebonden Onderzoek (CCMO) uitgevoerd”. If these policies are not in accordance, ZonMw may use article 19.4 of the Algemene Subsidiebepalingen ZonMw by adding extra conditions on royalties and extraction rights on databases created during the project.

If you have any questions after reading this letter, please contact the employee depicted in the header. If you are not satisfied with the way ZonMw has treated your application, you can let us know or make an official complaint (see below). Use in all correspondence the project number. Now that your application has been granted, the previous number is cancelled, and **the new project number is 848043004.**

Once again, we would like to congratulate you on this positive decision on your grant application. Good luck with the execution of your project.

Henk J. Smid, CEA ZonMw
